# Significance of Morpho-Palynological Diversity in Melliferous Plants in the Anzer Region (Türkiye) with Regard to Honey Authentication

**DOI:** 10.3390/plants14233600

**Published:** 2025-11-25

**Authors:** Zeynep Türker, Kamil Coşkunçelebi, Esra Demir Kanbur, Mutlu Gültepe

**Affiliations:** 1 Department of Biology, Faculty of Science, Karadeniz Technical University, 61080 Trabzon, Türkiye; zeynepturker@ktu.edu.tr (Z.T.);; 2 Central Research Laboratory Application and Research Center, Recep Tayyip Erdoğan University, 53100 Rize, Türkiye; 3 Department of Forestry, Dereli Vocational School, Giresun University, 28950 Giresun, Türkiye

**Keywords:** Anzer Honey, melliferous plants, microscopy, pollen characteristics

## Abstract

Morpho-palynological studies are essential to distinguish the botanical and geographical origins of honey, ensuring its authenticity, quality, and commercial value. This study examined 64 melliferous plant species (including 6 endemics) from the Anzer Valley to characterize pollen morphology using light and scanning electron microscopy. Of these, 26 taxa were analyzed morphologically for the first time. The evaluation of the results revealed that among the 21 flowering plant families identified, Fabaceae is represented by the highest number of taxa, followed by Asteraceae, Lamiaceae, and Rosaceae. Palynological findings showed that plants with medium-sized pollen grains are the most dominant, followed by those with small-sized pollen grains, while plants with large-sized pollen grains are present in the lowest proportion. At the same time, tricolporate (59% species) was represented by more than half of the examined species. Also, the microechinate–perforate type was the most dominating exine ornamentation, contributing 13% of the total ornamentation, while reticulate–perforate and striate–perforate represented 11% each, respectively. A generalized linear mixed-effects model (the polar axis as the response, the equatorial diameter as the predictor, the taxon as a random intercept) revealed that pollen size variation was primarily species-specific. While 41 species showed a positive trend, four exhibited a negative one, and 19 showed no clear association. The overall fixed-effect slope was moderately positive and statistically significant (*β* = 0.50 ± 0.02 SE, *p* < 0.001). These results emphasize the morphological diversity among taxa rather than a single allometric pattern.

## 1. Introduction

Melliferous plants are essential to the beekeeping profession as they provide nectar and pollen, a critical resource for honeybees and honey production. These plants, rich in nutritional compounds, support honeybee foraging activities and facilitate honey formation by domesticated bees [1]. Honeybees play a vital ecological and economic role, pollinating approximately 400 crop species and 16% of global flowering plants, which enhances agricultural productivity and maintains biodiversity [2,3]. Melliferous flora also holds ethnobotanical value, serving as sources of medicine, timber, livestock feed, and energy [4,5]. The symbiotic relationship between bees and plants ensures pollination and plant fertility while revealing honey’s botanical origin through pollen analysis. Understanding this interaction is key to advancing apiculture, assessing honey quality, and supporting global ecological balance.

Pollen morphology—specifically grain size, shape, and surface ornamentation—serves as a reliable indicator for identifying the plants foraged by bees [6]. Melliferous plants exhibit distinctive pollen traits that reflect the botanical and geographical origin of honey [7]. Beyond taxonomy, pollen morphology also reflects ecological and evolutionary adaptations. Traits such as polar axis length (P), equatorial diameter (E), and exine ornamentation are influenced by pollination syndrome, environmental conditions, and phylogenetic lineage [8,9]. Understanding these variations is therefore essential not only for honey authentication but also for linking plant-pollinator interactions with ecological gradients.

Advancements in image analysis and artificial neural networks have further enhanced the accuracy of pollen identification, thereby improving the authentication of honey’s botanical and geographical origins [10]. A comprehensive understanding of pollen morphology not only aids in verifying honey authenticity but also strengthens quality control in apicultural products, ensuring consumer trust and supporting sustainable beekeeping practices. Recent SEM-based morphometric studies have highlighted the diagnostic potential of fine-scale exine and aperture characters for honey-source determination, underscoring the relevance of quantitative LM/SEM datasets for melliferous flora [11,12]

Anzer (Ballıköy), situated near İkizdere in Rize, Türkiye, is renowned for its distinctive geography and ecological features. The alpine vegetation and diversity of flora, comprising over 500 flowering plant species, distinguish Anzer from other regions [13,14]. Anzer is one of Türkiye’s most unique apicultural landscapes because of its remarkable floral diversity, which directly affects the quality of the honey made there. The region’s high-altitude glacial valley environment, rich endemic flora, and cooperative-controlled Protected Geographical Indication (PGI) status [15] make Anzer Honey economically valuable and ecologically distinctive [16].

Türkiye, as one of the leading honey-producing countries, offers a remarkable diversity of honey types, each shaped by its distinct flora and geography [17]. Almost a quarter of the world’s 25 bee subspecies are found in Türkiye [18]. Of all the varied types of honeys (flower, pine, chestnut, sunflower, citrus, thyme, mad honey, etc.) produced in Türkiye, Anzer Honey, which is a multifloral honey, stands out in terms of its quality, price, and medicinal value. It is characterized by its dominant plant species from Fabaceae, alongside families like Apiaceae, Asteraceae, Boraginaceae, Rosaceae, Geraniaceae, and Lamiaceae without pollen grains from chestnut (*Castanea sativa* Mill.) and Komar (*Rhododendron ponticum* L.) plants [15]. According to Sorkun and Doğan [19], Fabaceae and *Trifolium* are the dominant family and genus for honey samples produced in Anzer Valley. Over the years, Anzer Honey has been studied for its physicochemical [20], and chemical [17,21,22,23] characteristics, while Türker et al. [24] studied its molecular characteristics.

Anzer Honey is exclusively produced by local cooperatives using nectar from these diverse and endemic plants [25]. Honey harvesting particularly occurs in the first two weeks of August, as *Apis mellifera* bees gather nectar during the limited flowering period between July and August, which is heavily influenced by climatic conditions. The short flowering period and altitude-specific flora give rise to a distinctive nectar composition that underpins the honey’s quality and authenticity.

These environmental and botanical factors are directly reflected in the chemical composition and therapeutic potential of Anzer Honey. The purity and composition of honey strongly influence its health benefits; these vary significantly depending on the geographical characteristics of the production region, local flora, harvest time, and production methods [26]. Anzer Honey, with its distinct regional attributes, is particularly notable for its high medicinal value. The pollen grains from plants in the Anzer Valley are exceptionally rich in vitamins, minerals, and proteins, contributing to their potent protective effects against various liver, digestive, and some skin diseases [27]. These unique qualities not only enhance the therapeutic properties of Anzer Honey but also drive high demand and premium pricing for this exceptional product [17,20].

In recent years, the production of artificial honey by feeding bees with sugar syrup has increased. Some producers produce artificial honey, which they market as natural honey by feeding bees too much sugar syrup during periods of high nectar flow. This leads to unfair competition. To distinguish between natural and artificial honey, in 2002, Sorkun and Doğan collected 127 natural honey and 44 artificial honey samples from different regions of Turkey and analyzed them for pollen. The study found that the total pollen count (TPN-10 gr) of honey is an important criterion in distinguishing between natural and artificial honey [28].

Despite studies addressing the physicochemical [17,20] and molecular [24,29] characteristics of Anzer Honey, the palynological diversity and quantitative morphological variation in the region’s melliferous flora remain insufficiently documented. Because pollen morphology reflects both phylogenetic structure and ecological adaptations, a comprehensive morpho-palynological dataset is essential for improving honey authentication. This represents a major gap for both palynological research and honey authentication in the eastern Black Sea region.

This study, therefore, aims to (1) characterize the pollen morphology of 64 melliferous plant species of the Anzer Valley (including 6 endemics) using LM and SEM, (2) quantify allometric relationships between polar and equatorial axes using a generalized linear mixed-effects model, and (3) identify morphological traits with potential diagnostic value for distinguishing taxa and supporting honey authentication efforts. By integrating classical palynology with quantitative modeling, the dataset expands the regional pollen reference and provides a baseline for future image-based classification approaches in honey authentication.

## 2. Materials and Methods

### 2.1. Study Area, Plant Collection, and Identification

**Figure 1:**
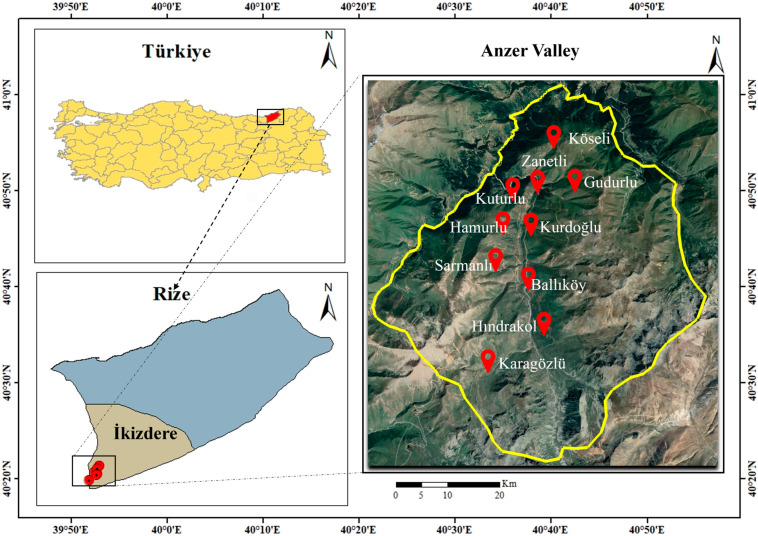
A combined map showing the regions where plant samples were collected in Anzer, Rize, Türkiye.

The study was conducted in ten localities within the Anzer Valley, Rize Province—Ballıköy, Gudurlu, Hamurlu, Hındrakol, Karagözlü, Köseli, Kurdoğlu, Kuturlu, Sarmanlı, and Zanetli (Figure 1). The valley, located at elevations between 1750–3150 m, hosts rich alpine vegetation and numerous endemic species [13,14]. The climate is typically humid and cool, with frequent rainfall and limited sunny days, conditions that support diverse melliferous flora and the production of the well-known Anzer Honey [14,15]. Annual precipitation and the average temperature are 2254.4 mm and 14.3 °C, respectively [30].

Various field visits between June and September were conducted to observe and collect the melliferous plant species from different locations in the Anzer Valley between 2022 and 2024. All plant material, including endemic taxa, was collected under fieldwork permissions granted by the local forestry directorate and in accordance with national regulations governing the collection of wild flora in Türkiye. A total of 64 plant species selected based on the field observations and preliminary interviews with the beekeepers, most frequently visited by honeybees, were collected and photographed, several of which were endemic to the Anzer region (Table 1). For each species, pollen grains were collected from at least three different individuals belonging to the same natural populations of Anzer Valley. Necessary information like date, collector number, habitat, coordinates, etc., was also recorded in the field notebook. The plant samples were brought to the plant taxonomy Lab of the Biology department at Karadeniz Technical University and dried according to herbarium techniques for identification and further analysis. All samples were identified according to Flora of Turkey and The East Aegean Islands [31,32] and other relevant literature [13,14]. Identified vouchers were stored in the Herbarium of Biology (KTUB) at Karadeniz Technical University (Table 1). Botanical names were validated using The Plant List, and Global Biodiversity Information Facility (GBIF) [33,34], and further confirmed with the checklist of Vascular Turkish Plants [35].

### 2.2. Palynological Studies

Flowers used in the present study were collected from natural habitats in the field. Pollen slides for light microscopy (LM) were prepared according to Erdtman, [36] acetolysis method as follows. Pollen and debris were separated by crushing anthers in 10% KOH, filtering, and centrifuging for 15 min at 3500 rpm. The pollen grains were treated with an acetolysis mixture of sulfuric acid and acetic anhydride, then boiled to remove contaminants while preserving the exine. After boiling, the pollen grains were thoroughly cleaned and stabilized by washing with distilled water, ethanol, and glycerin using a series of centrifuge operations. For each taxon, 2–3 acetolysed slides were prepared to observe and score palynological properties within the examined taxa. After that, they were permanently preserved by mounting them on slides using glycerin-gelatin and drying overnight for examination under a light microscope. The remaining pallet was preserved in 96% ethanol for scanning electron microscopy (SEM) studies.

Measurements and observations were made on 30 fully developed pollen grains per sample under the light microscope (Leica S6D (Leica Microsystems GmbH, Wetzlar, Germany). The choice of 30 pollen grains per sample was based on both statistical and practical considerations, as a sample size of 30 is widely recognized as sufficient for achieving reliable estimates under the central limit theorem, ensuring that sample means approximate a normal distribution [37]. Palynological features, including polar axis, equatorial diameter, the ratio of lengths of polar axis to the equatorial diameter (P/E), pollen type, shape, and surface ornamentation, were analyzed following the terminology of Halbritter et al. [9]. The shape of the pollen grains was determined by the ratio of P to E, and the size classes were indicated by the largest arithmetic mean of the diameter of pollen grains belonging to each species. As the pollen size varies from less than 10 μm to more than 100 μm, the size class was categorized as very small: <10 μm, small: 10–25 μm, medium: 26–50 μm, large: 51–100 μm, and very large: >100 μm [9]. For SEM analysis, pollen grains stored in ethanol were firstly air dried and then mounted on aluminum stubs with double-sided adhesive tape, coated with gold in a Sputter Coater (VG Microtech, Uckfield, UK), and examined under JEOL JSM-6510LV (JEOL Ltd., Tokyo, Japan) at 5–15 kV in the Central Research Laboratory of Recep Tayyip Erdogan University.

### 2.3. Statistical Analysis

To quantify the relationship between polar axis length and equatorial diameter while accounting for species-level variation, we fitted a generalized linear mixed-effects model (GLMM) with Gaussian errors using the statsmodels MixedLM framework (Python 3.13) [38]. Polar axis length (P) was the response variable, and equatorial diameter (E) was the fixed-effect predictor, with Taxon included as a random intercept to model species-specific deviations from the global mean:

(1) $$P_{ij}=\beta_{0}+\beta_{1}E_{ij}+u_{j}+\varepsilon_{ij}$$

where *P_ij_* represents the measured polar axis length of the *i-*th grain within the *j-*th species.

*β_0_* is the global intercept representing the mean pollen size across all species.

*β_1_* is the fixed-effect coefficient.

*E_ij_* is the fixed-effect variable.

*u_j_* represents the random intercept (taxon) for species, showing the deviations of each species’ mean from the global mean. Pooling pollen from multiple individuals per species ensured that the random intercept captured species-level rather than individual-level variation, thereby supporting the assumptions of the mixed-effects framework.

*ε_ij_* denotes the residual error for each observation.

The model assumed a Gaussian distribution because P and E are continuous and approximately normally distributed. Model fitting used maximum likelihood (ML) via the L-BFGS optimizer (Python 3.13). Both random-intercept and random-slope structures were evaluated, and model selection followed information-theoretic criteria (AIC) and likelihood-ratio tests to identify the most appropriate random-effects specification. Model fit summaries included fixed-effect significance, variance components, and marginal/conditional $R^{2}$ following Nakagawa & Schielzeth [39]. A random-slope extension was additionally fitted only to visualize species-specific allometric trends, while statistical inference was based on the final selected random-intercept model. Scatterplots with fitted lines were used to illustrate the relationships. Residual diagnostics were performed using quantile–quantile (Q–Q) plots to assess normality and residual-versus-fitted plots to evaluate homoscedasticity. Marginal and conditional $R^{2}$ values were computed using the variance-partitioning approach of Nakagawa & Schielzeth [39].

## 3. Results

### 3.1. Evaluation of Floristic and Pollen Morphology Findings

**Figure 2:**
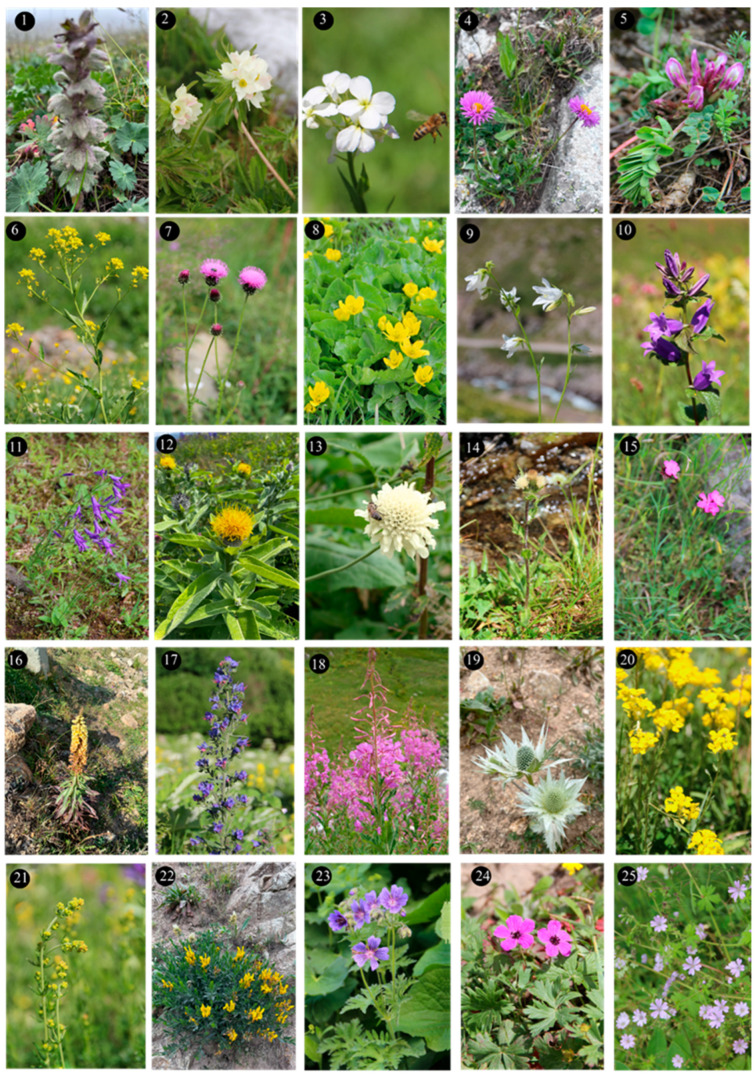
Floral field photographs of *Ajuga orientalis* L. (**1**), *Anemone narcissiflora* L. (**2**), *Arabis hirsute* (L.) Scop. (**3**), *Aster alpinus* L. (**4**), *Astragalus fricki* Bunge (**5**), *Bunias orientalis* L. (**6**), *Carduus adpressus* C.A.Mey. (**7**), *Caltha palustris* L. (**8**), *Campanula alliariifolia* Willd. (**9**), *Campanula glomerata* L. (**10**), *Campanula olympica* Boiss. (**11**), *Centaurea helenioides* Boiss. (**12**), *Cephalaria gigantea* (Ledeb.) Bobrov (**13**), *Cirsium pseudopersonata* Boiss. & Balansa (**14**), *Dianthus carmelitarum* Reut. ex Boiss. (**15**), *Digitalis ferruginea* L. (**16**), *Echium vulgare* L. (**17**), *Epilobium angustifolium* L. (**18**), *Eryngium giganteum* M.Bieb. (**19**), *Erysimum pulchellum* (Willd.) J. Gay (**20**), *Galium verum* L. (**21**), *Genista albida* Willd. (**22**), *Geranium ibericum* subsp. jubatum (Hand.-Mazz.) P.H.Davis (**23**), *Geranium ponticum* (P.H.Davis & J.Roberts) Aedo (**24**), *Geranium pyrenaicum* Burm.f. (**25**).

**Figure 3:**
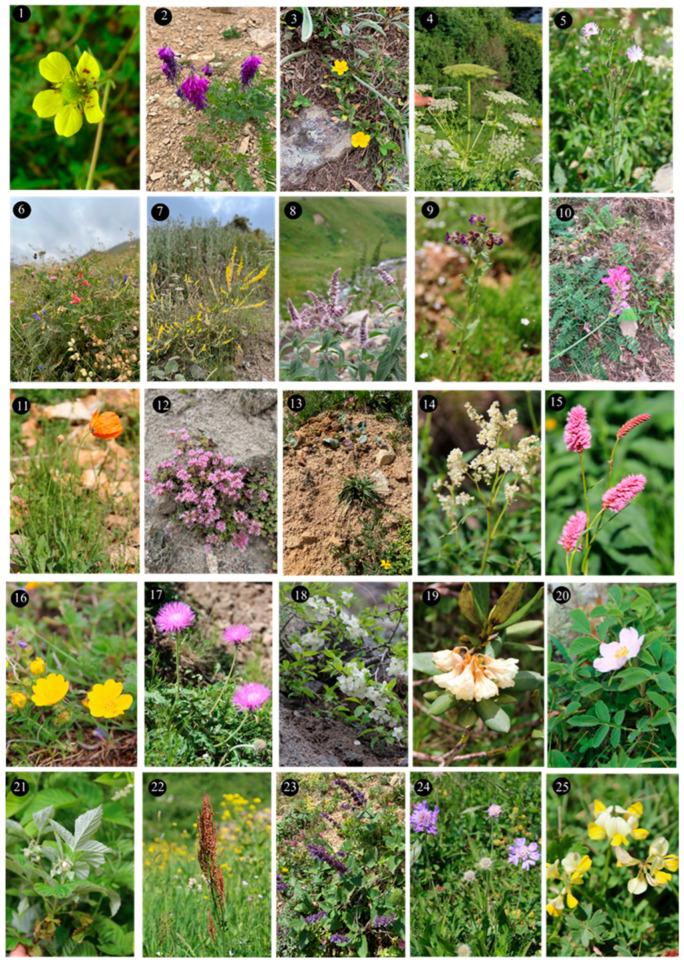
Floral field photographs of *Geum aleppicum* Jacq. (**1**), *Hedysarum hedysaroides* (L.) Schinz & Thell. (**2**), *Helianthemum nummularium* (L.) Mill. (**3**), *Heracleum platytaenium* Boiss. (**4**), *Lactuca racemose* Willd. (**5**), *Lathyrus roseus* Steven (**6**), *Melilotus officinalis* (L.) Desr. (**7**) *Mentha longifolia* (L.) L. (**8**), *Nonea versicolor* (Steven) Sweet (**9**), *Onobrychis altissima* Grossh. (**10**), *Papaver lateritium* K.Koch (**11**), *Phedimus spurius* (M.Bieb.) ’t Hart (**12**), *Plantago lanceolata* L. (**13**), *Polygonum alpinum* All. (**14**), *Polygonum bistorta* subsp. *carneum* (K.Koch) Coode & Cullen (**15**), *Potentilla crantzii* (Crantz) Fritsch (**16**), *Psephellus pulcherrimus* (Willd.) Wagenitz (**17**), *Prunus divaricate* Ledeb. (**18**), *Rhododendron caucasicum* Pall (**19**), *Rosa canina* L. (**20**), *Rubus idaeus* L. (**21**), *Rumex alpinus* L. (**22**), *Salvia verticillate* L. (**23**), *Scabiosa caucasica* M.Bieb. (**24**), *Securigera orientalis* (Mill.) Lassen (**25**).

**Figure 4:**
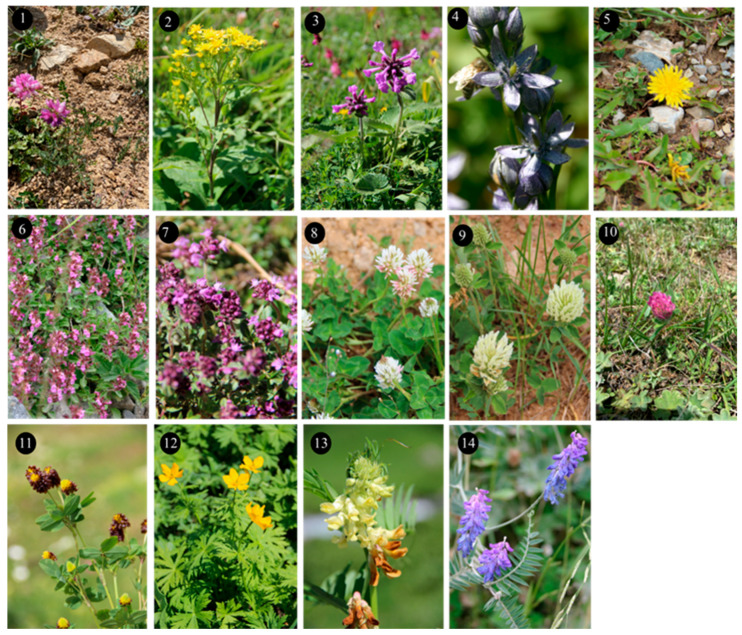
Floral field photographs of *Securigera varia* (L.) Lassen (**1**), *Solidago virgaurea* subsp. *alpestris* (Waldst. & Kit.) Gaudin (**2**), *Stachys macrantha* (K.Koch) Stearn (**3**), *Swertia iberica* Fisch. ex C.A.Mey. (**4**), *Taraxacum serotinum* (Waldst. & Kit.) Poir. (**5**), *Teucrium chamaedrys* L. (**6**), *Thymus praecox* Opiz (**7**), *Trifolium ambiguum* M.Bieb. (**8**), *Trifolium canescens* Willd. (**9**), *Trifolium pratense* L. (**10**), *Trifolium spadiceum* L. (**11**), *Trollius ranunculinus* (Sm.) Stearn (**12**), *Vicia balansae* Boiss. (**13**), *Vicia cracca* subsp. *cracca* L. (**14**).

**Figure 5:**
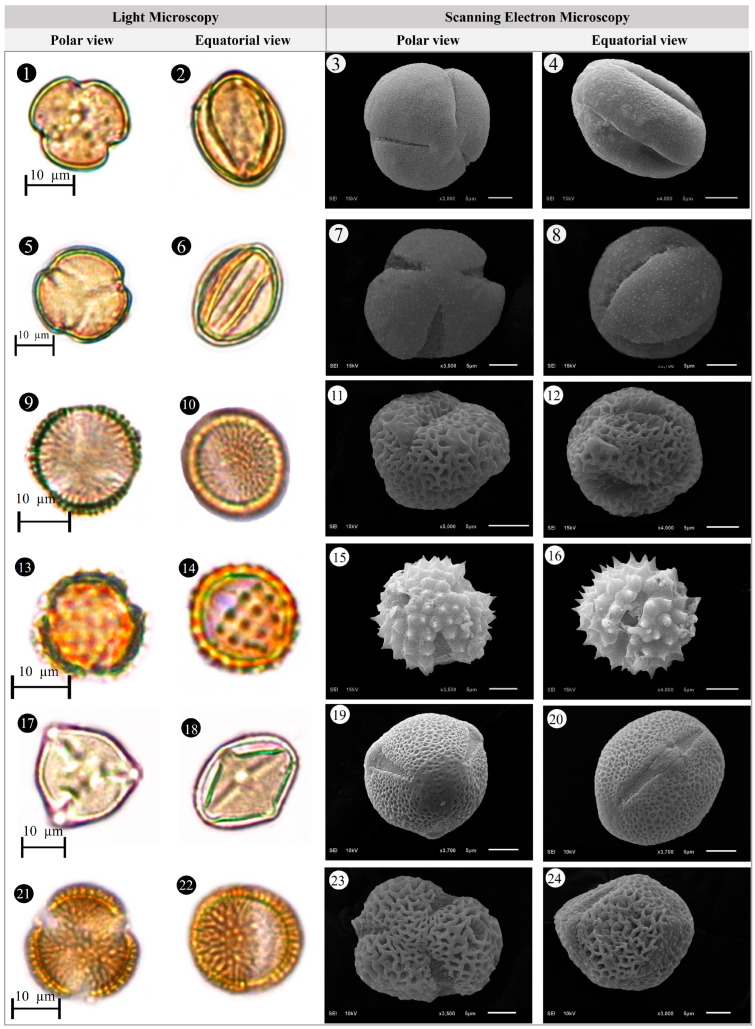
Pollen grains of *Ajuga orientalis* L. (**1**–**4**), *Anemone narcissiflora* L. (**5**–**8**), *Arabis hirsute* (L.) Scop. (**9**–**12**), *Aster alpinus* L. (**13**–**16**), *Astragalus fricki* Bunge (**17**–**20**), *Bunias orientalis* L. (**21**–**24**).

**Figure 6:**
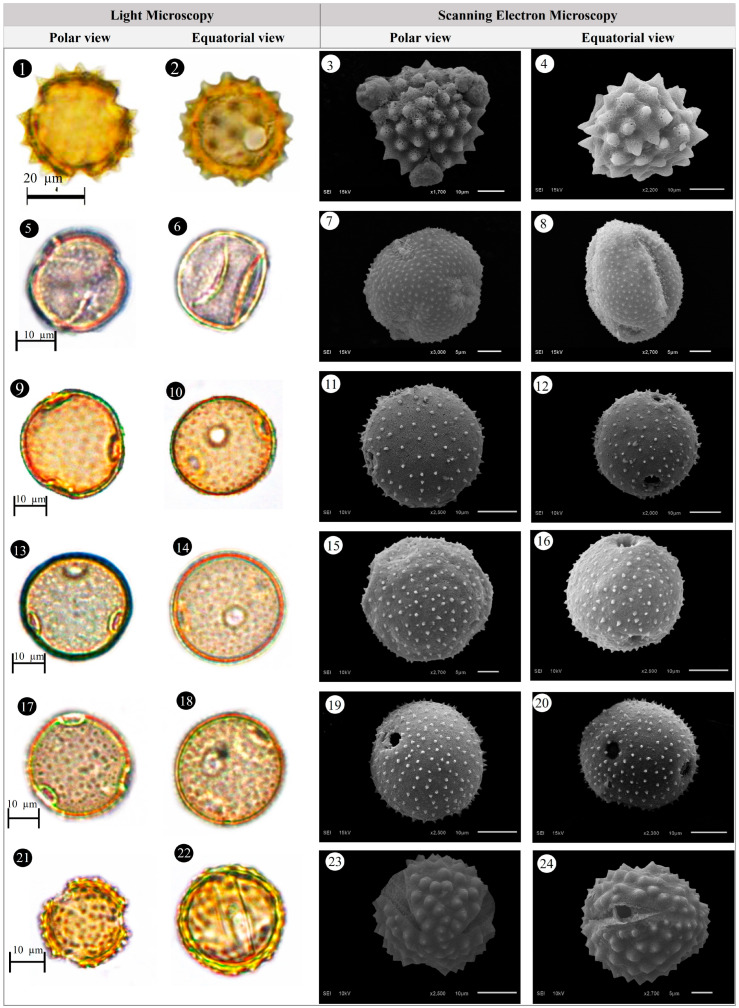
Pollen grains of *Carduus adpressus* C.A.Mey. (**1**–**4**), *Caltha palustris* L. (**5**–**8**), *Campanula alliariifolia* Willd. (**9**–**12**), *Campanula glomerata* L. (**13**–**16**), *Campanula olympica* Boiss. (**17**–**20**), *Centaurea helenioides* Boiss. (**21**–**24**).

**Figure 7:**
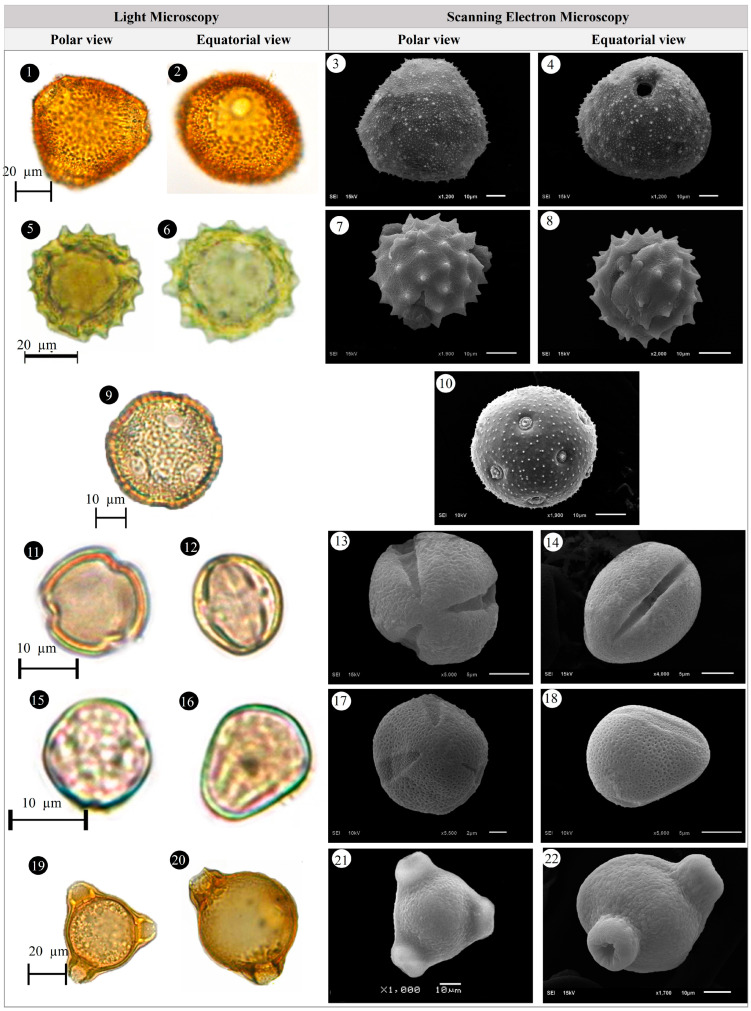
Pollen grains of *Cephalaria gigantea* (Ledeb.) Bobrov (**1**–**4**), *Cirsium pseudopersonata* Boiss. & Balansa (**5**–**8**), *Dianthus carmelitarum* Reut. ex Boiss. (**9**–**10**), *Digitalis ferruginea* L. (**11**–**14**), *Echium vulgare* L. (**15**–**18**), *Epilobium angustifolium* L. (**19**–**22**).

**Figure 8:**
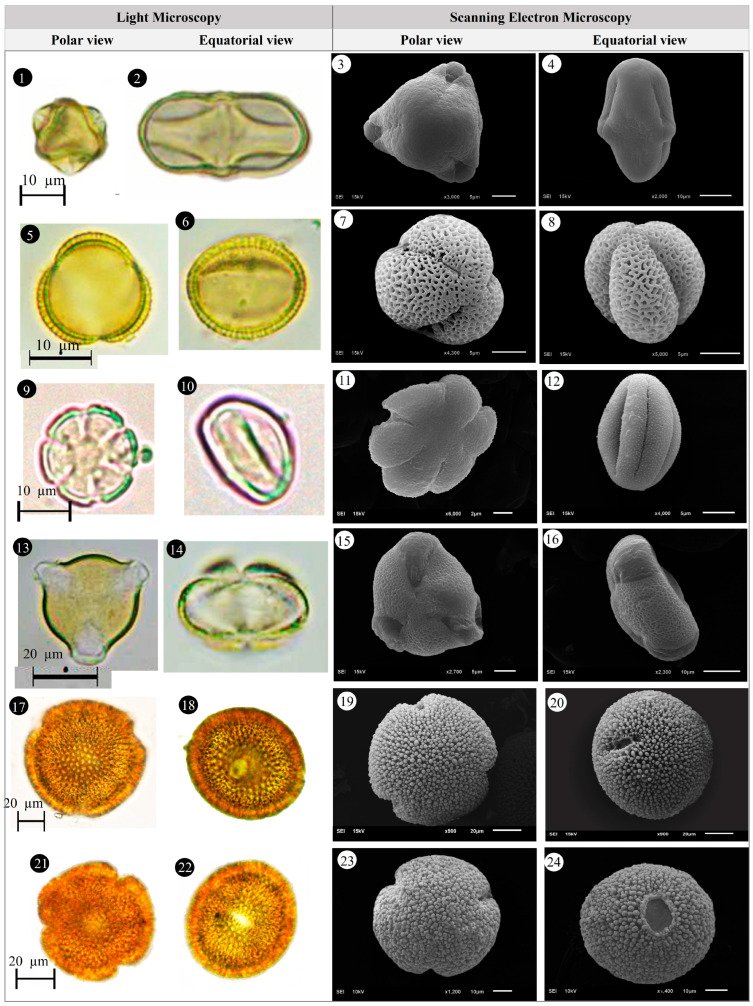
Pollen grains of *Eryngium giganteum* M.Bieb. (**1**–**4**), *Erysimum pulchellum* (Willd.) J. Gay (**5**–**8**), *Galium verum* L. (**9**–**12**), *Genista albida* Willd. (**13**–**16**), *Geranium ibericum* subsp. *jubatum* (Hand.-Mazz.) P.H.Davis (**17**–**20**), *Geranium ponticum* (P.H.Davis & J.Roberts) Aedo (**21**–**24**).

**Figure 9:**
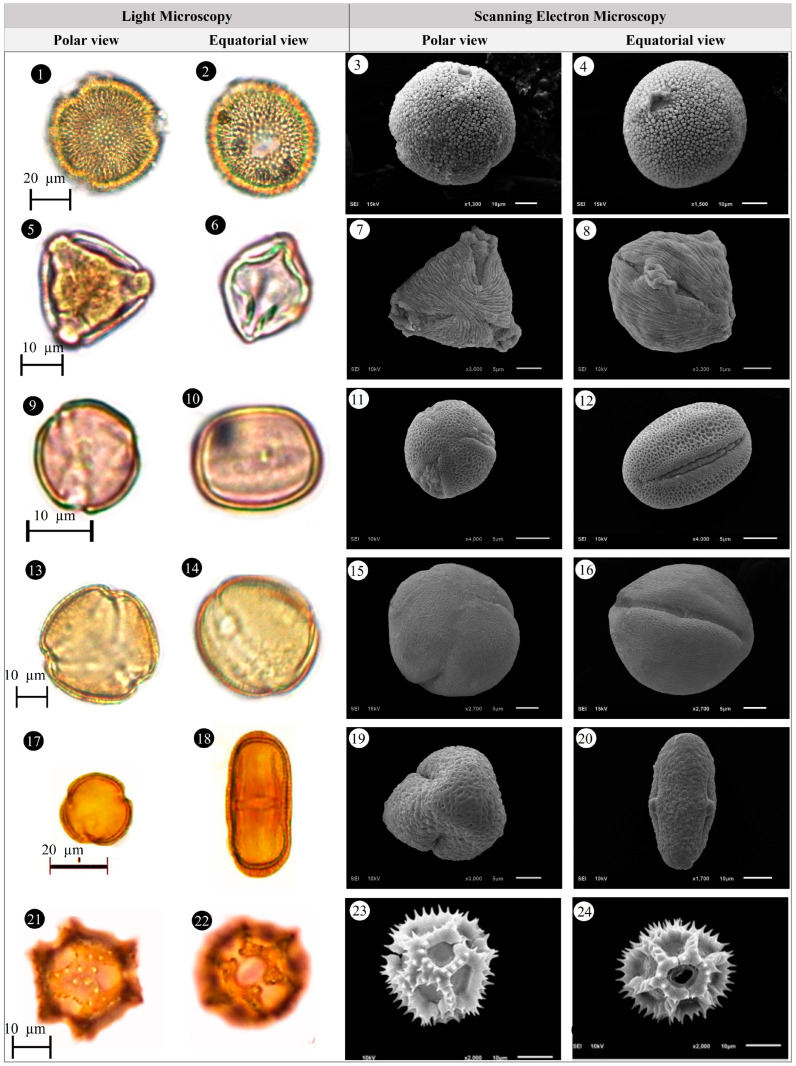
Pollen grains of *Geranium pyrenaicum* Burm.f. (**1**–**4**), *Geum aleppicum* Jacq. (**5**–**8**), *Hedysarum hedysaroides* (L.) Schinz & Thell. (**9**–**12**), *Helianthemum nummularium* (L.) Mill. (**13**–**16**), *Heracleum platytaenium* Boiss. (**17**–**20**), *Lactuca racemosa* Willd. (**21**–**24**).

**Figure 10:**
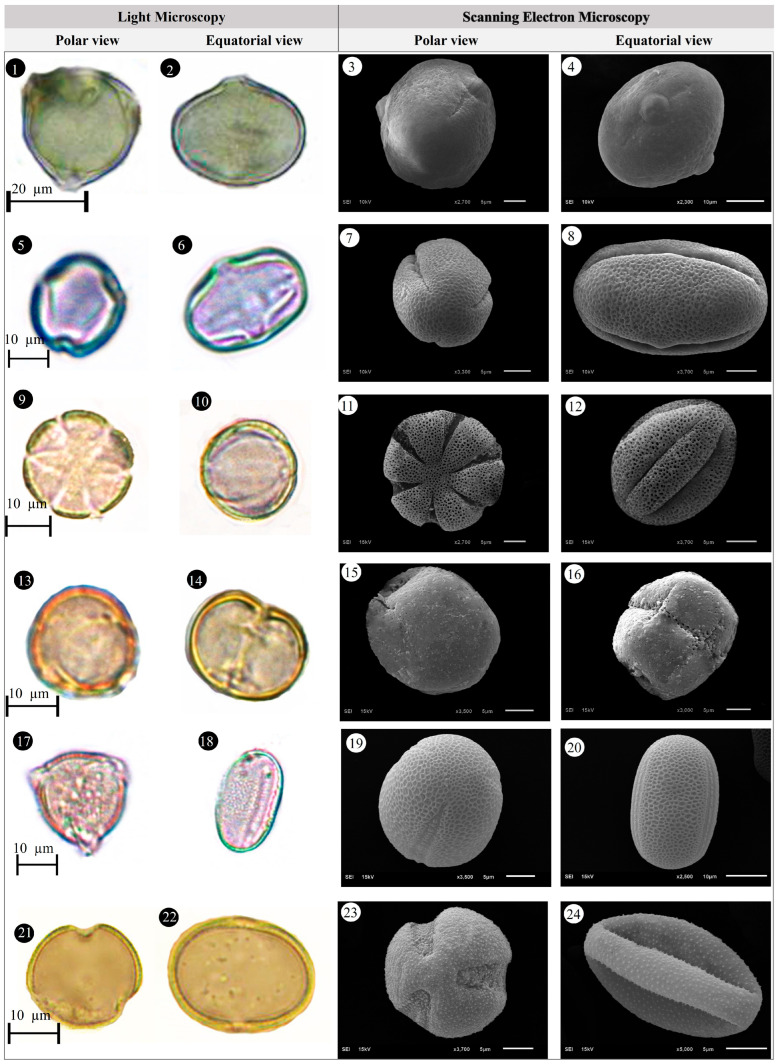
Pollen grains of *Lathyrus roseus* Steven (**1**–**4**), *Melilotus officinalis* (L.) Desr. (**5**–**8**), *Mentha longifolia* (L.) L. (**9**–**12**), *Nonea versicolor* (Steven) Sweet (**13**–**16**), *Onobrychis altissima* Grossh. (**17**–**20**), *Papaver lateritium* K.Koch (**21**–**24**).

**Figure 11:**
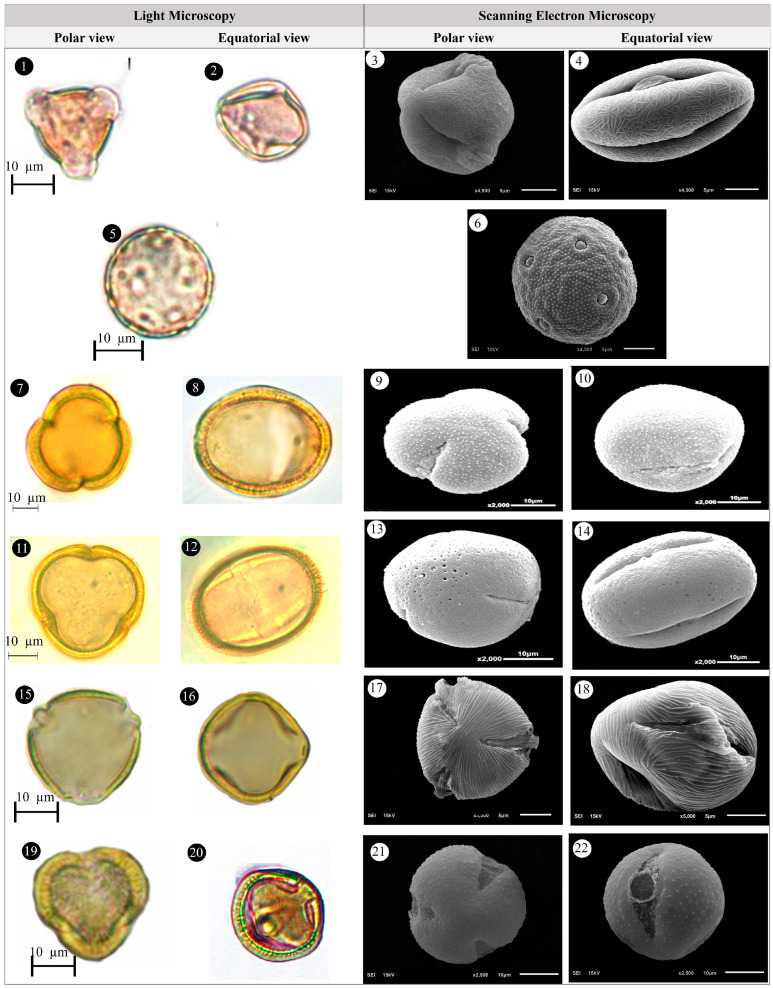
Pollen grains of *Phedimus spurius* (M.Bieb.) ’t Hart (**1**–**4**), *Plantago lanceolata* L. (**5**–**6**), *Polygonum alpinum* All. (**7**–**10**), *Polygonum bistorta* subsp. *carneum* (K.Koch) Coode & Cullen (**11**–**14**), *Potentilla crantzii* (Crantz) Fritsch (**15**–**18**), *Psephellus pulcherrimus* (Willd.) Wagenitz (**19**–**22**).

**Figure 12:**
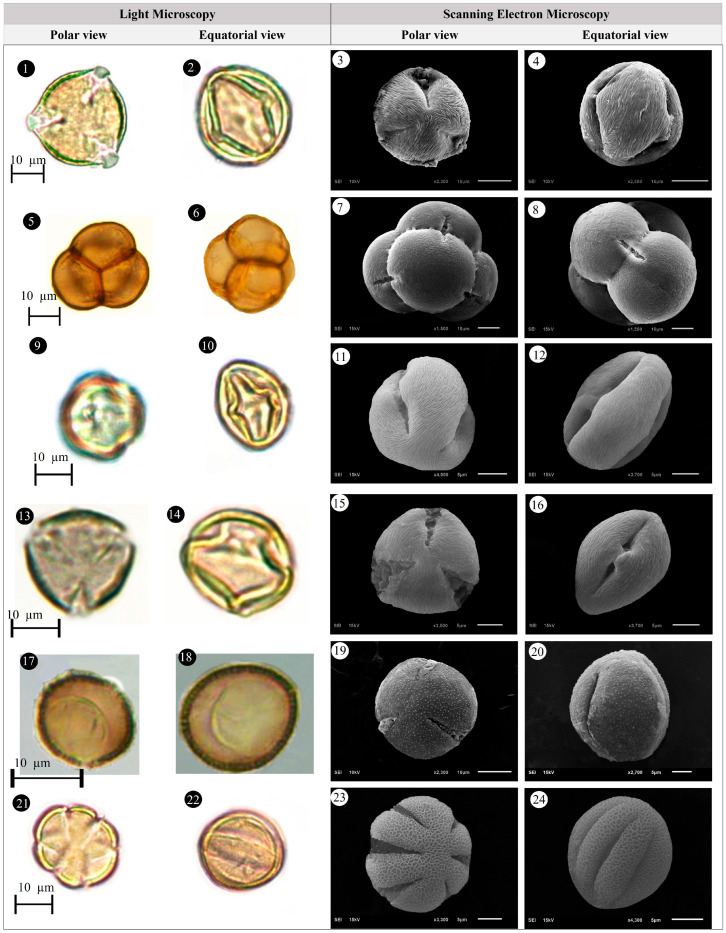
Pollen grains of *Prunus divaricate* Ledeb. (**1**–**4**), *Rhododendron caucasicum* Pall (**5**–**8**), *Rosa canina* L. (**9**–**12**), *Rubus idaeus* L. (**13**–**16**), *Rumex alpinus* L. (**17**–**20**), *Salvia verticillata* L. (**21**–**24**).

**Figure 13:**
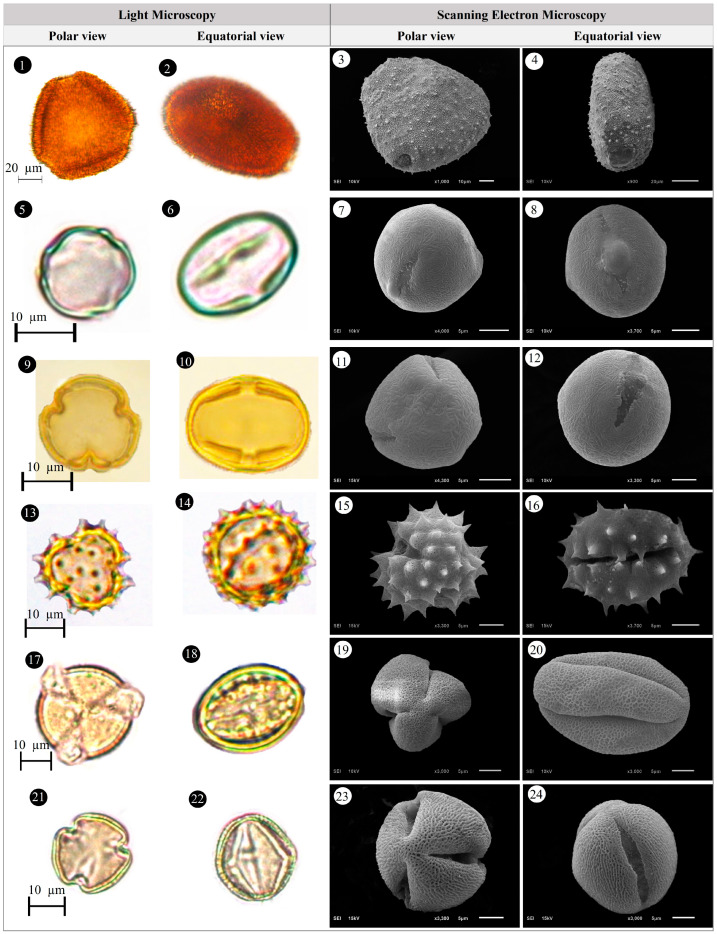
Pollen grains of *Scabiosa caucasica* M.Bieb. (**1**–**4**), *Securigera orientalis* (Mill.) Lassen (**5**–**8**), *Securigera varia* (L.) Lassen (**9**–**12**), *Solidago virgaurea* subsp. *alpestris* (Waldst. & Kit.) Gaudin (**13**–**16**), *Stachys macrantha* (K.Koch) Stearn (**17**–**20**), *Swertia iberica* Fisch. ex C.A.Mey. (**21**–**24**).

**Figure 14:**
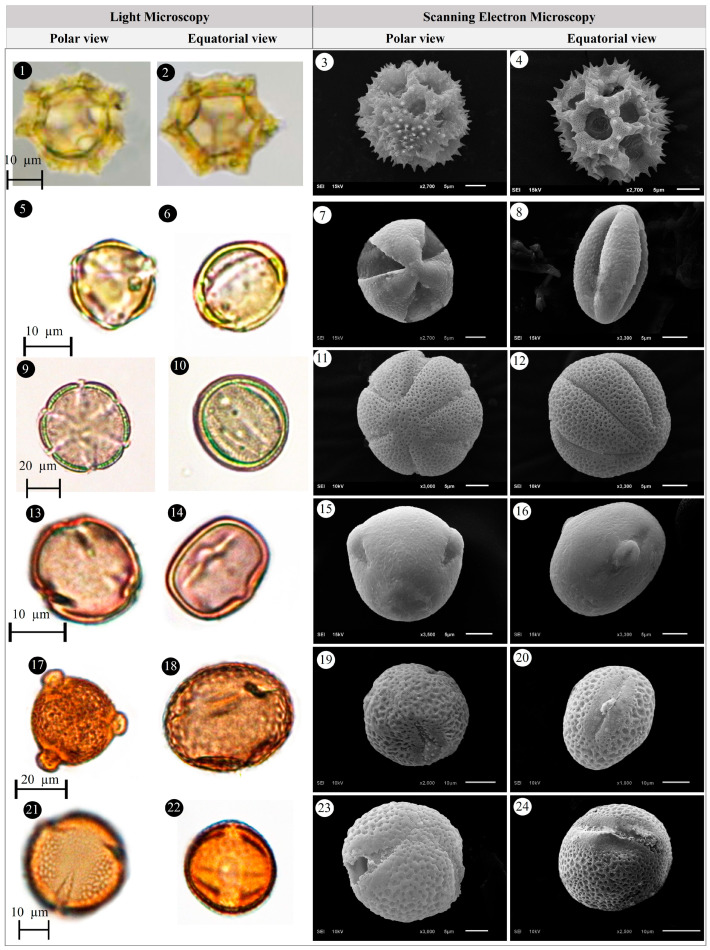
Pollen grains of *Taraxacum serotinum* (Waldst. & Kit.) Poir. (**1**–**4**), *Teucrium chamaedrys* L. (**5**–**8**), *Thymus praecox* Opiz (**9**–**12**), *Trifolium ambiguum* M.Bieb. (**13**–**16**), *Trifolium canescens* Willd. (**17**–**20**), *Trifolium pratense* L. (**21**–**24**).

**Figure 15:**
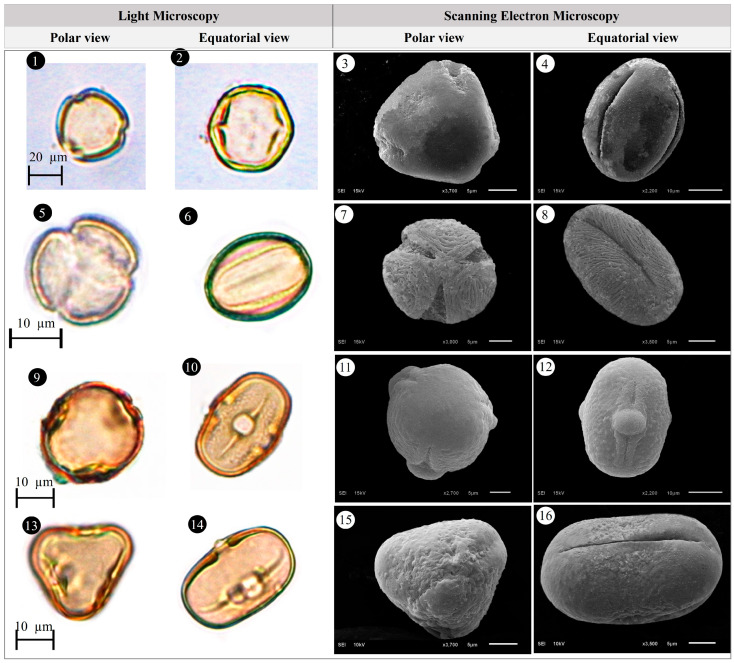
Pollen grains of *Trifolium spadiceum* L. (**1**–**4**), *Trollius ranunculinus* (Sm.) Stearn (**5**–**8**), Vicia balansae Boiss. (**9**–**12**), *Vicia cracca* subsp*. cracca* L. (**13**–**16**).

Within the scope of the present study, 64 melliferous plant species belonging to 21 families were collected and photographed from the Anzer Valley in light of both the personal observations and the information obtained from the beekeepers (Table 2, Figure 2, Figure 3 and Figure 4). The majority of the identified species (66%) belong to Fabaceae (14 species), followed by Asteraceae (8 species), Lamiaceae (6 species), and Rosaceae (5 species). It has been determined that the richest genera in terms of species number are *Trifolium* (4 species), *Campanula* (3 species), and *Geranium* (3 species). The examined morphological characters of the pollen grains of the selected melliferous plants, along with their polar and equatorial LM and SEM microphotographs, are presented in Table 2 and Figure 5, Figure 6, Figure 7, Figure 8, Figure 9, Figure 10, Figure 11, Figure 12, Figure 13, Figure 14 and Figure 15, respectively. Three different pollen shapes (spheroidal, oblate, and prolate) were determined among the examined taxa. Pollen types vary as hexacolpate, pantoporate, tetracolporate, tricolpate, tricolporate, triporate, and stephanocolpate. Twenty-four different types of pollen surface ornamentation were found in the examined species.

### 3.2. Polar Axis and Equatorial Diameter

**Figure 16:**
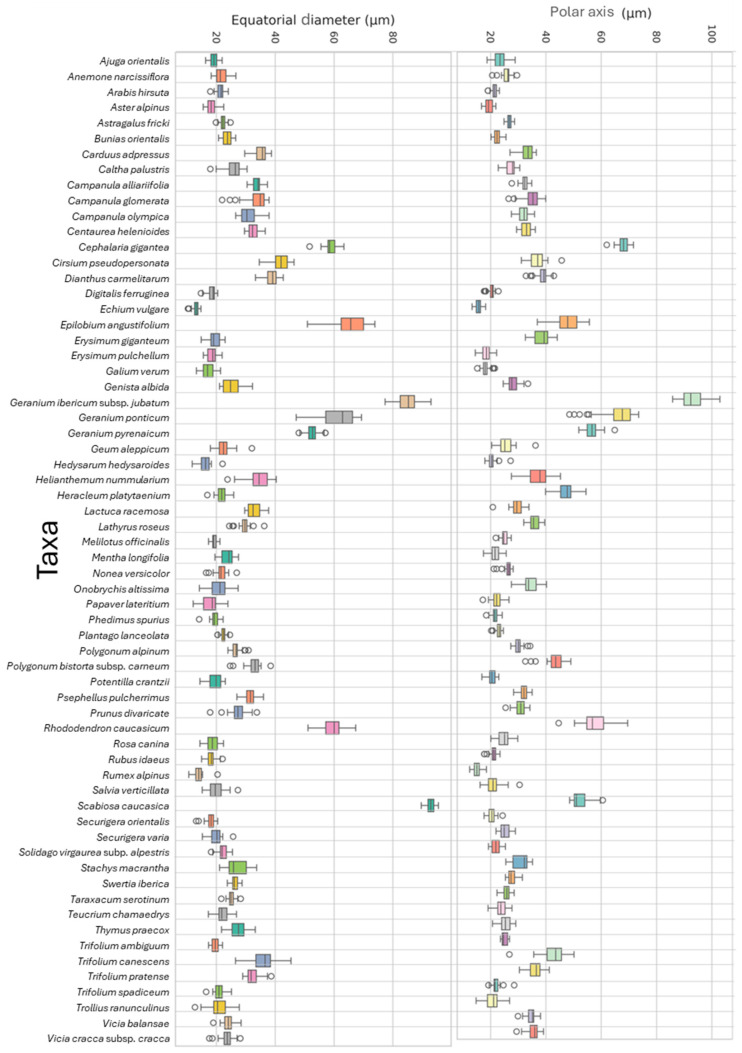
Box plot distribution of the Polar axis length and Equatorial diameter (in μm) of various melliferous plants in Anzer Valley.

A significant difference in the length of the polar axis and the equatorial diameter among the species investigated (Table 2) was observed. Figure 16 illustrates the box-plot results for P and E of the examined melliferous plants. The minimum average E value was recorded in *Echium vulgare*, while the maximum average E value was observed in *Scabiosa caucasica.* Similarly, the minimum and the maximum average *p* values are 15.06 ± 1.35 μm and 92.88 ± 4.18 μm, respectively, recorded in *Rumex alpinus* and *Geranium ibericum* subsp*. jubatum*, respectively.

### 3.3. Pollen Size

**Figure 17:**
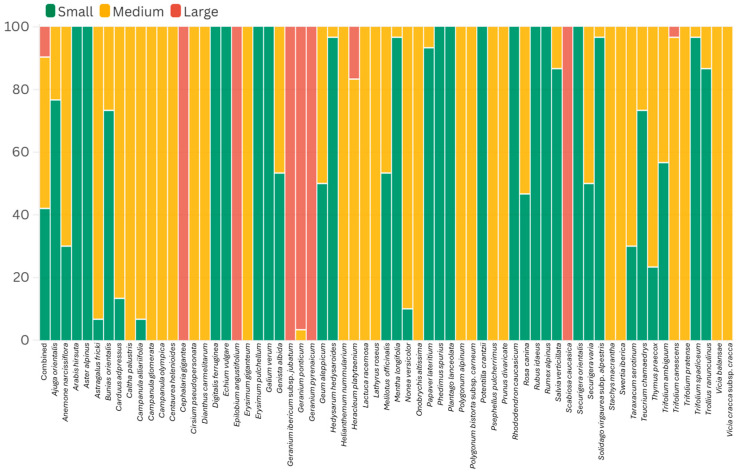
Pollen size categories of the 64 melliferous plants in Anzer Valley.

Medium-sized pollen showed the highest average representation of 47%, followed by small-sized pollen (42%) and large-sized pollen (11%), as shown in Figure 17. A total of 13 species were fully categorized as small, while 20 were medium, and only 5 species showed 100% representation for large. Also, the species predominantly represented by small-sized pollen were *Hedysarum hedysaroides*, *Mentha longifolia*, *Papaver lateritium*, *Solidago virgaurea* subsp. *alpestris*, and *Trifolium spadiceum.* Similarly, species predominantly represented by medium-sized pollen were *Astragalus fricki*, *Caltha palustris*, *Campanula glomerata*, *Nonea versicolor*, and *Trifolium canescens*, while *Geranium ponticum* was the only species mostly represented by large-sized pollen.

### 3.4. The Ratio of Polar Axis to Equatorial Diameter and Pollen Shape

The highest and the lowest values of P/E ratio for the selected melliferous plant species were 2.14 for *Heracleum platytaenium* and 0.55 for *Scabiosa caucasica,* respectively. The pollen grains were classified as prolate, oblate, and spheroidal based on the P/E ratio. As seen in Table 2, 57.81% (37 species) of the total species were prolate, 32.81% (21 species) were spheroidal, and the least represented pollen shape was oblate (9.38%; 6 species).

### 3.5. Pollen Type and Exine Ornamentation

**Figure 18:**
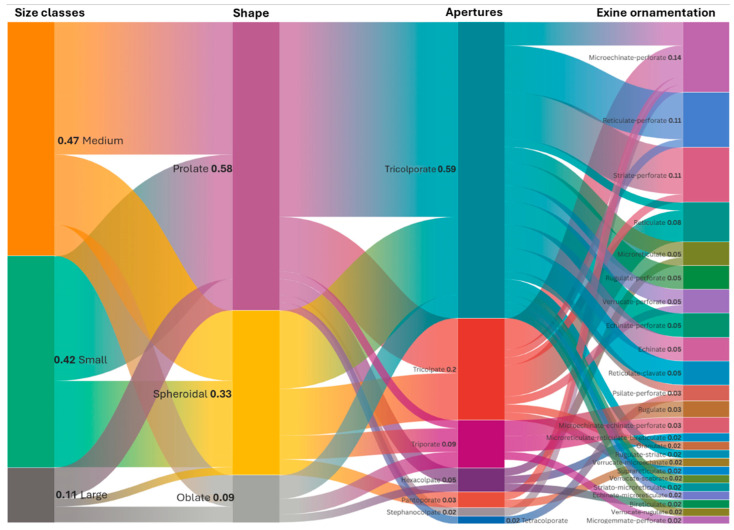
Alluvial plot showing the pollen size, shape, type, and ornamentation of the selected melliferous plants.

A total of 7 aperture types were identified from the 64 species, of which tricolporate constituted 59%, while tricolpate represented 20%. Stephanocolpate and tetracolporate were the least represented pollen types, constituting only 2% of the total aperture types each. On the other hand, a wide variety of exine ornamentations were found in the pollen grains. Microechinate-perforate was the most dominant ornamentation, accounting for 14% of the total of 24 different ornamentations. Figure 18 illustrates the different pollen sizes, shapes, types, and ornamentations of the examined samples.

### 3.6. Relationship Between Polar Axis and Equatorial Diameter

Model comparison showed differences between information-theoretic and likelihood-based criteria. The random-slope model had a lower AIC (AIC slope = 8662.33) than the random-intercept model (AIC intercept = 8948.97), but the likelihood-ratio test indicated that the additional random-slope term did not significantly improve model fit (LR = 267.41, df = 2, *p* = 0.47). Because the random-slope variance was small and the LR test did not support the added complexity, the random-intercept model was retained for inference, while the random-slope extension was used only to visualize species-specific allometric trajectories.

A GLMM fitted with polar axis as the response and equatorial diameter as the fixed effect revealed a strong size relationship across taxa. The fixed-effect slope was significantly positive (*β* = 0.50 ± 0.02 SE, z = 25.1, *p* < 0.001), indicating that pollen grains tend to elongate along the polar axis as equatorial diameter increases. The model explained a substantial proportion of the variance (marginal R^2^ = 0.46, conditional R^2^ = 0.96). Species-level random intercepts were highly variable (variance = 65.6), reflecting strong interspecific differences in mean pollen size (Table 3). The residual variance was 5.0. Residual diagnostics indicated no major violations of normality or homoscedasticity, confirming that the Gaussian mixed-effects model was appropriate for the data.

**Figure 19:**
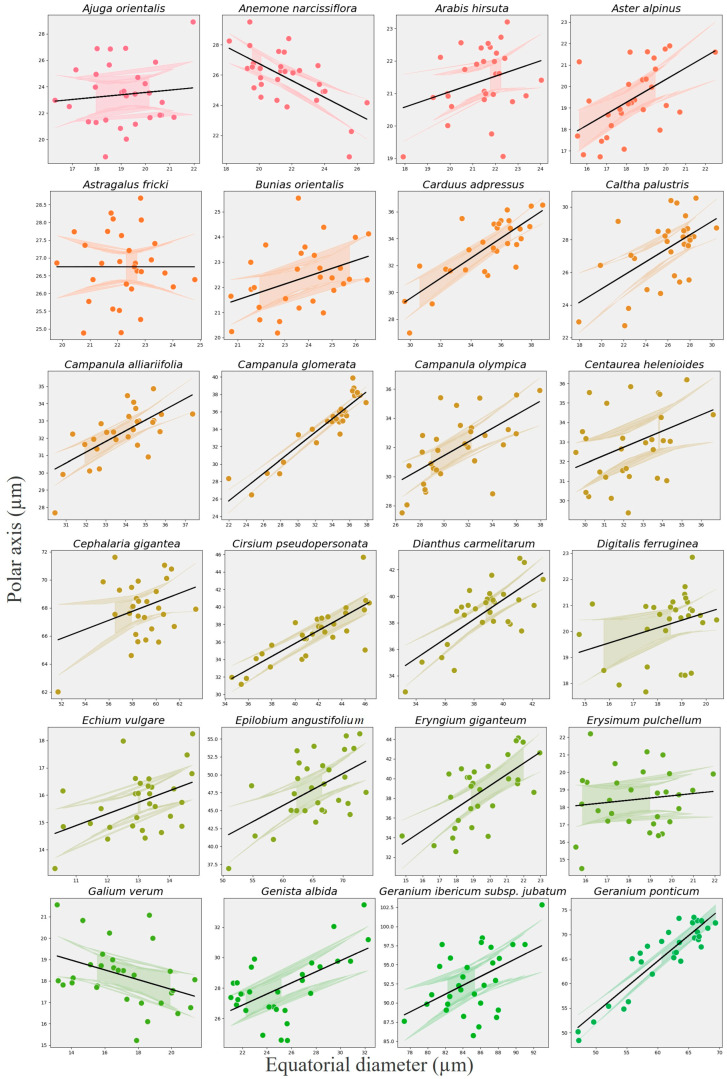
Species-specific scatterplots showing the relationship between polar axis (P) and equatorial diameter (E) for the first 24 melliferous plant species in alphabetical order. Black lines show the overall GLMM fixed-effect trend, while colored lines (with 95% CI) show species-level exploratory slopes for 24 melliferous species in alphabetical order.

**Figure 20:**
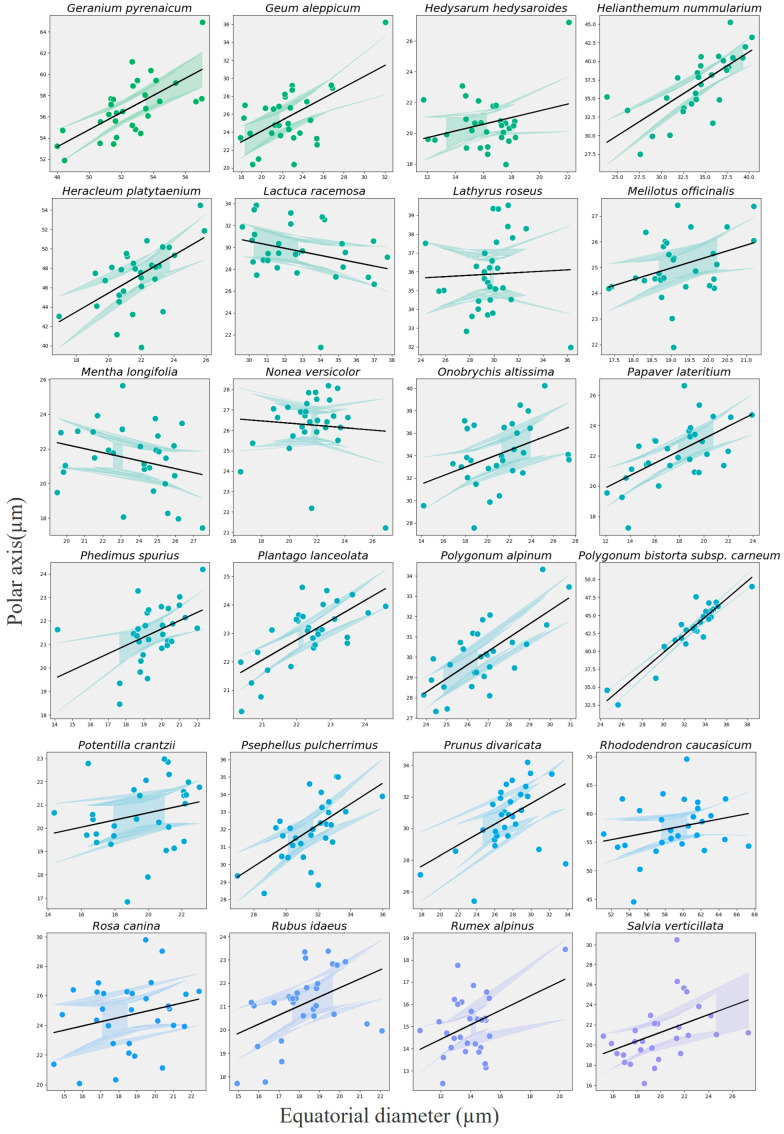
Species-specific scatterplots showing the relationship between polar axis (P) and equatorial diameter (E) for the subsequent 24 melliferous plant species in alphabetical order. Black lines show the overall GLMM fixed-effect trend, while colored lines (with 95% CI) show species-level exploratory slopes for 24 melliferous species in alphabetical order.

**Figure 21:**
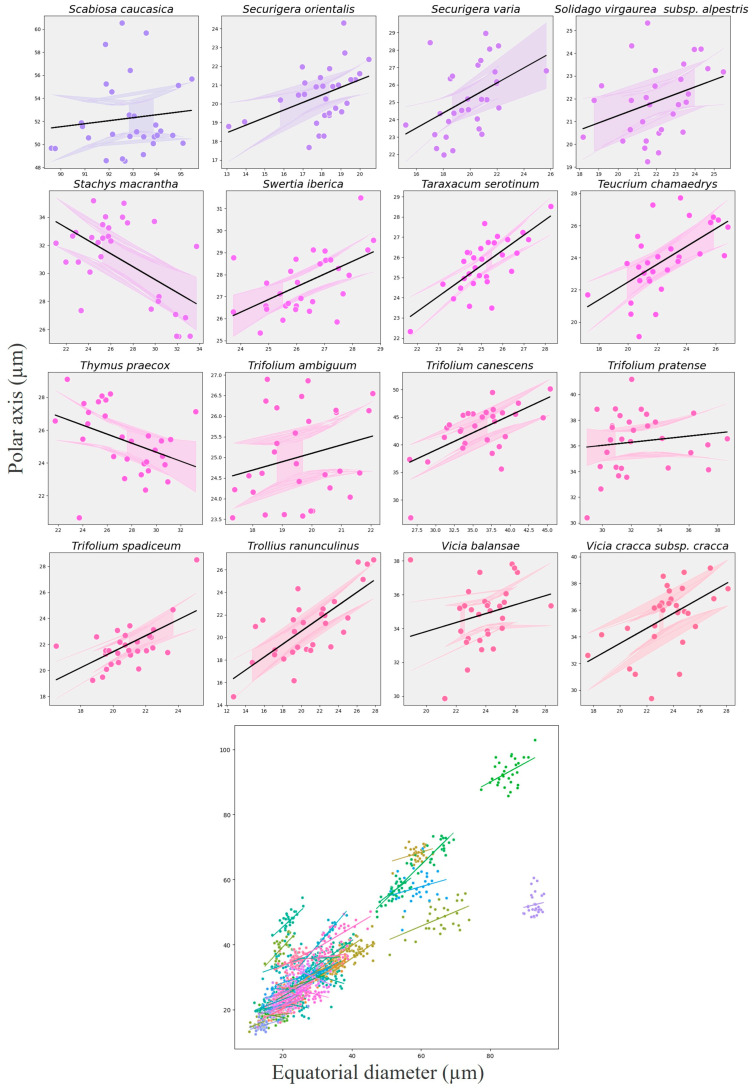
Overview of the relationship between polar axis (P) and equatorial diameter (E) across 64 species. Upper panels show species-specific scatterplots with GLMM fixed-effect slope (black) and exploratory species-level slopes (colored). The lower combined panel overlays all species, illustrating the global trend and interspecific variation.

Species-specific trends, visualized using a random-slope extension for exploratory purposes, showed considerable heterogeneity: 41 species displayed a positive P–E association, four species (e.g., *Anemone narcissiflora*, *Galium verum*, *Stachys macrantha*, *Thymus praecox*) showed a negative trend, and 19 taxa exhibited no clear relationship (Figure 19, Figure 20 and Figure 21). These patterns indicate that allometric scaling varies among taxa, even though the overall fixed effect is strongly positive.

## 4. Discussion

This study examined 64 melliferous plant species from the Anzer Valley, including 6 endemics and 26 taxa analyzed morphologically for the first time. These new records expand the regional pollen reference and contribute to botanical source identification for Anzer Honey, which relies on a distinctive, high-altitude, and endemic-rich flora. Three (*Geranium ibericum* subsp. *jubalatum*, *Geranium ponticum*, and *Papaver lateratium*) of the six endemic species are medically invaluable [40].

The floristic composition of Anzer Valley, dominated by Fabaceae, Asteraceae, and Lamiaceae, aligns with previous regional studies [13,19] and reflects the preference of honeybees for these families. Previous research conducted by Güner et al. [13] and Sorkun & Doğan, [19] in the Anzer region (İkizdere) also identified Fabaceae and Asteraceae as the most important nectar sources for honeybees. The preference of honeybees for these plants has been associated with their relatively high protein content. Studies suggest that pollinators favor protein-rich pollen as it provides essential amino acids needed for larval nutrition and overall colony health. The same researchers also reported that plant species with a higher protein content are more likely to attract a larger number of pollinators [41,42]. Hotaman [27] documented elevated proline concentrations in Anzer Honey, which generally reflect protein-rich floral resources and may contribute to the nutritional relevance of these plants for foraging bees. These plant species have further shown to possess very high total phenolic content (44.07–124.10 mg/g pollen) in terms of content as well as variety, increasing the nutritional value of Anzer pollen [21].

Various species of Lamiaceae and Asteraceae (e.g., *Teucrium chamaedrys*, *Mentha longifolia*) contain essential oils and phenolic compounds such as rosmarinic acid, caffeic acid, and flavonoids [43], which may influence pollinator visitation patterns.

When comparing the pollen sizes, the distribution suggests that most melliferous plants in the Anzer region produce pollen that falls within the small-to-medium range. Medium-sized pollen grains were the most commonly observed category in this study, aligning with findings from previously published research [44,45]. However, the size of the pollen is found to have a minimal effect on the plant-bee interaction, according to García-García et al. [46], but it is still unclear if pollen grain size is controlled indirectly by trade-offs in pollen quantity or directly by pollinator foraging behaviors [47]. The predominance of medium-sized pollen in the region may be consistent with the local climatic conditions, which resemble those described in ecological models linking temperature regimes with pollen size distribution [48]. Medium-sized pollen has been suggested to represent a balance between dispersal potential and structural robustness, although the functional implications remain subject to further investigation. The temperate and occasionally windy climate of the region may also permit some degree of wind-mediated pollen movement, although the primary pollination mode for most taxa remains insect-based. From an applied perspective, the predominance of these size classes suggests that size alone will be a weak discriminator for authentication; instead, size should be combined with aperture and ornamentation traits in any identification model.

Analysis of the P/E ratio revealed that prolate pollen was the most common (58%), followed by spheroidal (33%) and oblate pollen (9%). The predominance of prolate pollen points to an overall entomophilous syndrome in the local flora, favoring adherence to insects and effective transfer. The relatively low proportion of spheroidal pollen is congruent with the region’s humid climate and the dominance of insect pollination rather than wind-pollination. Numerous studies have found that spheroidal pollens are typically found in dry regions because of their lower surface-to-volume ratios, which help retain water [48]. The relatively low frequency of spheroidal pollen grains may reflect the predominance of insect-pollinated taxa in this humid montane environment. Furthermore, since spheroidal is commonly associated with anemophilous pollination [45,49], the higher prevalence of prolate-shaped pollen in this study could suggest a greater dependence on entomophilous pollination.

Recent palynological studies from neighboring regions also show patterns comparable to the present findings. For example, pollen morphometrics reported from Eastern Anatolia, the Caucasus, and the broader Irano-Turanian floristic zone commonly document a dominance of prolate, tricolporate pollen grains with reticulate or echinate ornamentation in melliferous taxa, similar to the Anzer flora [44,45,47,50]. Studies on honey-source plants from Iran, Pakistan, and the Balkans further highlight Fabaceae, Asteraceae, and Lamiaceae as families with characteristic reticulate–perforate or echinate exine patterns used in honey authentication datasets [51,52,53]. Comparative SEM-based surveys from high-altitude or alpine floras (e.g., Northern Iran, Caucasus, and Eastern Turkey) also report medium-to-large pollen sizes and dominant tricolporate apertures, indicating that the Anzer Valley flora largely aligns with regional morphological trends while still exhibiting locally distinctive allometric variation and ornamentation diversity. This comparison supports the broader relevance of the Anzer pollen dataset for both regional floristics and global honey-source reference libraries.

Across the investigated taxa, the pollen data mainly support an entomophilous strategy. Medium-sized grains, tricolporate apertures, and diverse echinate, reticulate–perforate, or rugulate ornamentations were common in families such as Apiaceae, Asteraceae, Fabaceae, and Lamiaceae. These characteristics are common to insect-pollinated plants since the rough exine and well-defined apertures help adherence to the bodies of visiting insects, hence enhancing efficient pollen distribution. The literature has repeatedly seen such traits as adaptations improving pollinator specificity and efficiency [8]. Differences in pollen shape—whether spheroidal, prolate, or oblate—also align with patterns observed in other insect-pollinated montane floras. Families like Fabaceae and Lamiaceae, with prolate pollen grains and reticulate, rugulate–perforate, or microreticulate ornamentations, highlight the adaptations to insect pollination, as highlighted from the previous literature as well [50,51,54]. Even though the overall traits of the studied melliferous plants may be associated with entomophily, species like *Plantago lanceolata* with small, spheroidal pollen grains and microechinate ornamentation are beneficial for anemophilous pollination, as emphasized by Lu, Ye, and Liu [49].

Among the examined melliferous plant species, tricolporate and tricolpate pollen apertures were most prevalent, accounting for 59% and 20% of the total, respectively. Laallam et al. [45] reported that the prevalence of tricolporate and tricolpate pollen in honey plants helps in understanding and assessing their pollination needs, reproductive efficiency, and ecological relationships with pollinators, especially bees. Structural features of the tricolporate and tricolpate pollen grains generally facilitate pollen dispersal and germination during pollination. This structural adaptation further reinforces their dependence on entomophilous pollination, particularly by bees [45]. The prevalence of tricolporate and triporate apertures aligns with patterns commonly reported for many insect-pollinated species, although the functional significance of these aperture types requires further study [55,56].

The most prevalent ornamentation patterns among the plant species were microechinate-perforate (13% of the species), reticulate-perforate (11% of the species), and striate-perforate (11% of the species). All species of the Asteraceae, Caprifoliaceae, Rubiaceae, and 3 species of the Polygonaceae had echinate ornamentation. This type of ornamentation has been shown to enhance pollen adhesion to the bodies of the bees, facilitating effective transport. These observations about echinate ornamentation align with the findings of a study by Khan et al. [44]. Similarly, families like Fabaceae, Lamiaceae, and Brassicaceae predominantly possessed reticulate ornamentations, as is evident from other previous works [52,53]. It has been reported that pollen-reticulated surface ornamentation, which facilitates the adhesion of pollen to the bees’ bodies, may also facilitate the effective transport of pollen [57,58].

The GLMM demonstrated that most variation in pollen size arises from species-specific differences rather than from a uniform allometric scaling pattern across the flora. Although the overall P–E relationship was strongly positive (*β* = 0.50, *p* < 0.001), species displayed diverse trends, with positive, negative, or absent associations. This heterogeneity, supported by the large random-intercept variance, underscores the pronounced morphological diversity among Anzer taxa. The species-level trends, derived from an exploratory random-slope visualization, further emphasize that pollen dimensions do not follow a single allometric pathway but instead reflect lineage-specific morphological strategies.

The combined model (Figure 21) displays distinct morphological clusters representing small-sized (*Mentha longifolia*, *Echium vulgare*), medium-sized (*Eryngium giganteum*, *Centaurea helenioides*), and large-sized taxa (*Cephalaria gigantea*, *Geranium ponticum*). From a honey authentication perspective, this model-based differentiation among taxa provides a quantitative framework for distinguishing pollen assemblages in genuine Anzer Honey. Since each species exhibits a distinct allometric signature, integrating these size relationships into digital pollen reference datasets or automated image-based classification systems could improve predictive accuracy in melissopalynological authentication.

In order to improve the quality and productivity of Anzer Honey and to protect it against fraud, the floristic diversity of the Anzer Valley should be thoroughly studied as a preliminary step. This dataset may provide useful background information for regional apicultural management, although its direct application will require integration with ecological and phenological data. It is recommended that stakeholders and beekeepers in the apiculture industry prioritize the conservation and maintenance of melliferous plant diversity surrounding bee apiaries to improve nutritional value and quality, while also implementing microscopic analysis of honey for quality and authenticity control. The dataset can be used as a regional pollen reference and for immediate practical purposes, such as (i) microscopic screening of Anzer Honey for expected family-level pollen signatures, (ii) development of multivariate or image-based classifiers that are trained on combined LM/SEM features, and (iii) protecting key nectar sources.

While this study offers a thorough palynological examination of the Anzer Valley flora, it is important to recognize numerous limitations. The GLMM utilized to determine P–E correlations successfully accounted for interspecific variability; however, it excluded potential environmental factors such as altitude, soil moisture, or temperature gradients. Incorporating such variables in future analyses would help determine whether the observed morphometric variation reflects ecological or adaptive processes rather than solely taxonomic differences.

The implications for honey authentication, although exciting, remain speculative unless substantiated by actual honey samples. It is necessary to have a database that connects the detected pollen morphotypes to validated Anzer Honey samples in order to turn these morphometric correlations into useful tools for authentication. Adding these records to machine-learning classifiers or automated pollen recognition systems would make honey origin verification even more objective and scalable.

## 5. Conclusions

The present study provides a comprehensive morpho-palynological characterization of 64 melliferous plant species in the Anzer region, a globally recognized area for its geographically labeled honey. Pollen grains of these taxa exhibit substantial variation in size, aperture type, and exine ornamentation, reflecting both the taxonomic diversity of the local flora and their ecological adaptations to the region’s high-altitude environment.

These traits represent potential diagnostic markers requiring validation with actual honey samples, as emphasized by the strong allometric relationship between the polar axis and equatorial diameter (fixed-effect slope = 0.50; P/E ratio range = 0.55–2.14; conditional R^2^ = 0.96). Such quantitative descriptors highlight the morphological distinctiveness of Anzer flora and suggest promising avenues for improving honey authentication.

Expanding the number of taxa sampled—particularly those most frequently visited by honeybees—and conducting plant collections across an even broader portion of the vegetation period would further strengthen the reliability of the regional palynological reference. Integrating these expanded datasets with validated honey sediments or DNA metabarcoding results will provide a more rigorous basis for distinguishing genuine Anzer Honey from adulterated or mislabeled products.

Overall, the dataset generated in this study establishes a robust foundation for future taxonomic, ecological, and honey authentication research in the Anzer Valley and supports the development of multidisciplinary tools for verifying the botanical origin of this highly valued honey.

## Figures and Tables

**Table 1:** Collection information of the selected 64 melliferous plant species of Anzer Valley, Rize, Türkiye.

Family	Taxa	Locality	Altitude (m)	Voucher Number
Apiaceae	*Eryngium giganteum* M.Bieb.	İkizdere, Anzer, Ballıköy	2169	Gültepe & Z.Türker 285 (KTUB)
Apiaceae	*Heracleum platytaenium* Boiss.	İkizdere, Anzer, Hındrakol	2265	Coşkunçelebi & Z.Türker 81 (KTUB)
Asteraceae	*Aster alpinus* L.	İkizdere, Anzer, Ballıköy	2706	Gültepe & Z.Türker 267 (KTUB)
Asteraceae	*Carduus adpressus* C.A.Mey.	İkizdere, Anzer, Hındrakol	2190	Coşkunçelebi & Z.Türker 109 (KTUB)
Asteraceae	*Centaurea helenioides* Boiss.	İkizdere, Anzer, Kurdoğlu	2487	Gültepe & Z.Türker 258 (KTUB)
Asteraceae	*Cirsium pseudopersonata* Boiss. & Balansa	İkizdere, Anzer, Hındrakol	2416	Coşkunçelebi & Z.Türker 151 (KTUB)
Asteraceae	*Lactuca racemosa* Willd.	İkizdere, Anzer, Ballıköy	2136	Coşkunçelebi & Z.Türker 158 (KTUB)
Asteraceae	*Psephellus pulcherrimus* (Willd.) Wagenitz	İkizdere, Anzer, Hındrakol	2190	Coşkunçelebi & Z.Türker 114 (KTUB)
Asteraceae	*Solidago virgaurea* subsp*. alpestris* (Waldst. & Kit.) Gaudin	İkizdere, Anzer, Hındrakol	2416	Coşkunçelebi & Z.Türker 140 (KTUB)
Asteraceae	*Taraxacum serotinum* (Waldst. & Kit.) Poir.	İkizdere, Anzer, Hındrakol	2221	Coşkunçelebi & Z.Türker 13 (KTUB)
Boraginaceae	*Echium vulgare* L.	İkizdere, Anzer, Hındrakol	2238	Coşkunçelebi & Z.Türker 186 (KTUB)
Boraginaceae	*Nonea versicolor* (Steven) Sweet	İkizdere, Anzer, Hındrakol	2265	Coşkunçelebi & Z.Türker 73 (KTUB)
Brassicaceae	*Arabis hirsuta* (L.) Scop.	İkizdere, Anzer, Hındrakol	2265	Coşkunçelebi & Z.Türker 38 (KTUB)
Brassicaceae	*Bunias orientalis* L.	İkizdere, Anzer, Karagözlü	2460	Coşkunçelebi & Z.Türker 131 (KTUB)
Brassicaceae	*Erysimum pulchellum* (Willd.) J.Gay	İkizdere, Anzer, Hındrakol	2265	Coşkunçelebi & Z.Türker 39 (KTUB)
Campanulaceae	*Campanula alliariifolia* Willd.	İkizdere, Anzer, Hamurlu	2455	Coşkunçelebi & Z.Türker 137 (KTUB)
Campanulaceae	*Campanula glomerata* L.	İkizdere, Anzer, Hamurlu	2455	Coşkunçelebi & Z.Türker 138 (KTUB)
Campanulaceae	*Campanula olympica* Boiss.	İkizdere, Anzer, Köseli	1933	Coşkunçelebi & Z.Türker 129 (KTUB)
Caprifoliaceae	*Cephalaria gigantea* (Ledeb.) Bobrov	İkizdere, Anzer, Sarmanlı	2518	Gültepe & Z.Türker 246 (KTUB)
Caprifoliaceae	*Scabiosa caucasica* M.Bieb.	İkizdere, Anzer, Ballıköy	2136	Coşkunçelebi & Z.Türker 154 (KTUB)
Caryophyllaceae	*Dianthus carmelitarum* Reut. ex Boiss.	İkizdere, Anzer, Karagözlü	2460	Coşkunçelebi & Z.Türker 132 (KTUB)
Cistaceae	*Helianthemum nummularium* (L.) Mill.	İkizdere, Anzer, Hındrakol	2280	Gültepe & Z.Türker 253 (KTUB)
Crassulaceae	*Phedimus spurius* (M.Bieb.) ’t Hart	İkizdere, Anzer, Hındrakol	2587	Gültepe & Z.Türker 230 (KTUB)
Ericaceae	*Rhododendron caucasicum* Pall	İkizdere, Anzer, Hındrakol	2238	Coşkunçelebi & Z.Türker 185 (KTUB)
Fabaceae	*Astragalus fricki* Bunge	İkizdere, Anzer, Kuturlu	2108	Coşkunçelebi & Z.Türker 19 (KTUB)
Fabaceae	*Genista albida* Willd.	İkizdere, Anzer, Ballıköy	2148	Gültepe & Z.Türker 284 (KTUB)
Fabaceae	*Hedysarum hedysaroides* (L.) Schinz & Thell.	İkizdere, Anzer, Ballıköy	2650	Gültepe & Z.Türker 264 (KTUB)
Fabaceae	*Lathyrus roseus* Steven	İkizdere, Anzer, Ballıköy	2156	Gültepe & Z.Türker 282 (KTUB)
Fabaceae	*Melilotus officinalis* (L.) Desr.	İkizdere, Anzer, Ballıköy	2156	Gültepe & Z.Türker 283 (KTUB)
Fabaceae	*Onobrychis altissima* Grossh.	İkizdere, Anzer, Hındrakol	2341	Gültepe & Z.Türker 273 (KTUB)
Fabaceae	*Securigera orientalis* (Mill.) Lassen	İkizdere, Anzer, Kurdoğlu	2487	Gültepe & Z.Türker 259 (KTUB)
Fabaceae	*Securigera varia* (L.) Lassen	İkizdere, Anzer, Sarmanlı	2416	Coşkunçelebi & Z.Türker 170 (KTUB)
Fabaceae	*Trifolium ambiguum* M.Bieb.	İkizdere, Anzer, Hındrakol	2341	Gültepe & Z.Türker 275 (KTUB)
Fabaceae	*Trifolium canescens* Willd.	İkizdere, Anzer, Sarmanlı	2518	Gültepe & Z.Türker 244 (KTUB)
Fabaceae	*Trifolium pratense* L.	İkizdere, Anzer, Sarmanlı	2518	Gültepe & Z.Türker 248 (KTUB)
Fabaceae	*Trifolium spadiceum* L.	İkizdere, Anzer, Hındrakol	2190	Coşkunçelebi & Z.Türker 110 (KTUB)
Fabaceae	*Vicia balansae* Boiss.	İkizdere, Anzer, Hındrakol	2265	Coşkunçelebi & Z.Türker 54 (KTUB)
Fabaceae	*Vicia cracca* subsp*. cracca* L.	İkizdere, Anzer, Hındrakol	2115	Coşkunçelebi & Z.Türker 187 (KTUB)
Gentianaceae	*Swertia iberica* Fisch. ex C.A.Mey.	İkizdere, Anzer, Hındrakol	2239	Coşkunçelebi & Z.Türker 203 (KTUB)
Geraniaceae	*Geranium ibericum* subsp*. jubatum* (Hand.-Mazz.) P.H.Davis	İkizdere, Anzer, Hındrakol	2265	Coşkunçelebi & Z.Türker 77 (KTUB)
Geraniaceae	*Geranium ponticum* (P.H.Davis & J.Roberts) Aedo	İkizdere, Anzer, Hındrakol	2626	Coşkunçelebi & Z.Türker 87 (KTUB)
Geraniaceae	*Geranium pyrenaicum* Burm.f.	İkizdere, Anzer, Hamurlu	2455	Coşkunçelebi & Z.Türker 125 (KTUB)
Lamiaceae	*Ajuga orientalis* L.	İkizdere, Anzer, Ballıköy	2567	Gültepe & Z.Türker 270 (KTUB)
Lamiaceae	*Mentha longifolia* (L.) L.	İkizdere, Anzer, Hındrakol	2238	Coşkunçelebi & Z.Türker 180 (KTUB)
Lamiaceae	*Salvia verticillata* L.	İkizdere, Anzer, Sarmanlı	2518	Gültepe & Z.Türker 249 (KTUB)
Lamiaceae	*Stachys macrantha* (K.Koch) Stearn	İkizdere, Anzer, Kurdoğlu	2096	Coşkunçelebi & Z.Türker 96 (KTUB)
Lamiaceae	*Teucrium chamaedrys* L.	İkizdere, Anzer, Hındrakol	2238	Coşkunçelebi & Z.Türker 182 (KTUB)
Lamiaceae	*Thymus praecox* Opiz	İkizdere, Anzer, Hındrakol	2190	Coşkunçelebi & Z.Türker 105 (KTUB)
Onagraceae	*Epilobium angustifolium* L.	İkizdere, Anzer, Hındrakol	2238	Coşkunçelebi & Z.Türker 179 (KTUB)
Papaveraceae	*Papaver lateritium* K.Koch	İkizdere, Anzer, Ballıköy	2536	Gültepe & Z.Türker 272 (KTUB)
Plantaginaceae	*Digitalis ferruginea* L.	İkizdere, Anzer, Karagözlü	2460	Coşkunçelebi & Z.Türker 135 (KTUB)
Plantaginaceae	*Plantago lanceolata* L.	İkizdere, Anzer, Sarmanlı	2518	Gültepe & Z.Türker 243 (KTUB)
Polygonaceae	*Polygonum alpinum* All.	İkizdere, Anzer, Hındrakol	2265	Coşkunçelebi & Z.Türker 50 (KTUB)
Polygonaceae	*Polygonum bistorta* subsp*. carneum* (K.Koch) Coode & Cullen	İkizdere, Anzer, Hındrakol	2265	Coşkunçelebi & Z.Türker 52 (KTUB)
Polygonaceae	*Rumex alpinus* L.	İkizdere, Anzer, Kurdoğlu	2096	Coşkunçelebi & Z.Türker 94 (KTUB)
Ranumculaceae	*Anemone narcissiflora* L.	İkizdere, Anzer, Kuturlu	2108	Coşkunçelebi & Z.Türker 22 (KTUB)
Ranumculaceae	*Caltha palustris* L.	İkizdere, Anzer, Kuturlu	2108	Coşkunçelebi & Z.Türker 21 (KTUB)
Ranumculaceae	*Trollius ranunculinus* (Sm.) Stearn	İkizdere, Anzer, Hındrakol	2265	Coşkunçelebi & Z.Türker 58 (KTUB)
Rosaceae	*Geum aleppicum* Jacq.	İkizdere, Anzer, Hındrakol	2248	Gültepe & Z.Türker 281 (KTUB)
Rosaceae	*Potentilla crantzii* (Crantz) Fritsch	İkizdere, Anzer, Karagöz	2412	Coşkunçelebi & Z.Türker 06 (KTUB)
Rosaceae	*Prunus divaricata* Ledeb.	İkizdere, Anzer, Kuturlu	2108	Coşkunçelebi & Z.Türker 20 (KTUB)
Rosaceae	*Rosa canina* L.	İkizdere, Anzer, Hındrakol	2190	Coşkunçelebi & Z.Türker 102 (KTUB)
Rosaceae	*Rubus idaeus* L.	İkizdere, Anzer, Hındrakol	2190	Coşkunçelebi & Z.Türker 101 (KTUB)
Rubiaceae	*Galium verum* L.	İkizdere, Anzer, Hındrakol	2238	Coşkunçelebi & Z.Türker 175 (KTUB)

**Table 2:** Palynological characteristics of the investigated melliferous plants distributed in Anzer Valley (Based on SEM observations). *: Represents the taxa examined for the first time in the present paper; †: Represents the endemic species; SD: Standard deviation.

Family	Taxa	P ± SD (μm)	E ± SD (μm)	P/E	Shape	Pollen Size	Aperture	Exine Ornamentation
Apiaceae	*Eryngium giganteum* M.Bieb. *	38.89 ± 3.72	19.40 ± 2.08	2	Prolate	Medium	Tricolporate	Verrucate–scabrate
Apiaceae	*Heracleum platytaenium* Boiss.	47.02 ± 3.63	21.98 ± 2.07	2.14	Prolate	Medium	Tricolporate	Rugulate–perforate
Asteraceae	*Aster alpinus* L.	19.42 ± 1.58	18.30 ± 1.62	1.06	Spheroidal	Small	Tricolporate	Echinate-perforate
Asteraceae	*Carduus adpressus* C.A.Mey.	33.32 ± 2.27	35.04 ± 2.39	0.95	Spheroidal	Medium	Tricolporate	Echinate
Asteraceae	*Centaurea helenioides* Boiss. *†	33.05 ± 2.05	32.59 ± 1.94	1.01	Spheroidal	Medium	Tricolporate	Echinate
Asteraceae	*Cirsium pseudopersonata* Boiss. & Balansa †	36.91 ± 3.11	41.47 ± 3.43	0.89	Oblate	Medium	Tricolporate	Echinate
Asteraceae	*Lactuca racemosa* Willd.	29.91 ± 3.55	32.93 ± 3.68	0.91	Oblate	Medium	Tricolporate	Echinate–microreticulate
Asteraceae	*Psephellus pulcherrimus* (Willd.) Wagenitz *	31.95 ± 1.72	31.47 ± 1.75	1.02	Spheroidal	Medium	Tricolporate	Microechinate-perforate
Asteraceae	*Solidago virgaurea* subsp*. alpestris* (Waldst. & Kit.) Gaudin *	21.89 ± 1.57	22.11 ± 1.74	0.99	Spheroidal	Small	Tricolporate	Echinate–perforate
Asteraceae	*Taraxacum serotinum* (Waldst. & Kit.) Poir.	24.86 ± 1.33	24.84 ± 1.26	1	Spheroidal	Small	Tricolporate	Echinate–perforate
Boraginaceae	*Echium vulgare* L.	15.76 ± 1.11	13.05 ± 1.19	1.21	Prolate	Small	Tricolporate	Reticulate–perforate
Boraginaceae	*Nonea versicolor* (Steven) Sweet *	26.26 ± 1.55	22.53 ± 2.79	1.17	Prolate	Medium	Tetracolporate	Psilate–perforate
Brassicaceae	*Arabis hirsuta* (L.) Scop.	21.38 ± 1.04	21.37 ± 1.30	1	Spheroidal	Small	Tricolpate	Reticulate
Brassicaceae	*Bunias orientalis* L.	22.35 ± 1.29	23.71 ± 1.66	0.94	Spheroidal	Small	Tricolpate	Reticulate
Brassicaceae	*Erysimum pulchellum* (Willd.) J.Gay *	18.43 ± 1.73	18.33 ± 1.74	1.01	Spheroidal	Small	Tricolpate	Reticulate
Campanulaceae	*Campanula alliariifolia* Willd. *	32.41 ± 1.61	33.96 ± 1.58	0.95	Spheroidal	Medium	Triporate	Rugulate
Campanulaceae	*Campanula glomerata* L.	33.55 ± 3.90	34.78 ± 3.32	0.96	Spheroidal	Medium	Triporate	Rugulate
Campanulaceae	*Campanula olympica* Boiss.	31.11 ± 3.04	31.95 ± 2.30	0.97	Spheroidal	Medium	Triporate	Rugulate
Caprifoliaceae	*Cephalaria gigantea* (Ledeb.) Bobrov *	68.02 ± 2.12	58.79 ± 2.17	1.16	Prolate	Large	Triporate	Microechinate–echinate–perforate
Caprifoliaceae	*Scabiosa caucasica* M.Bieb.	51.27 ± 1.62	92.84 ± 1.12	0.55	Oblate	Large	Triporate	Microechinate–echinate–perforate
Caryophyllaceae	*Dianthus carmelitarum* Reut. ex Boiss. †	38.92 ± 2.31	38.83 ± 2.22	1	Spheroidal	Medium	Pantoporate	Microechinate–perforate
Cistaceae	*Helianthemum nummularium* (L.) Mill. *	36.94 ± 4.13	34.7 ± 3.92	1.08	Prolate	Medium	Tricolporate	Striate–perforate
Crassulaceae	*Phedimus spurius* (M.Bieb.) ’t Hart	25.29 ± 1.32	19.18 ± 1.54	1.32	Prolate	Small	Tricolporate	Rugulate–striate
Ericaceae	*Rhododendron caucasicum* Pall *	57.59 ± 5.64	59.10 ± 4.51	0.97	Spheroidal	Large	Tricolporate	Verrucate–Rugulate
Fabaceae	*Astragalus fricki* Bunge *	26.86 ± 1.12	22.29 ± 1.15	1.21	Prolate	Medium	Tricolporate	Reticulate
Fabaceae	*Genista albida* Willd.	25.09 ± 3.99	28.83 ± 1.88	0.87	Oblate	Medium	Tricolporate	Microreticulate
Fabaceae	*Hedysarum hedysaroides* (L.) Schinz & Thell.	20.62 ± 1.71	16.22 ± 2.16	1.27	Prolate	Small	Tricolpate	Reticulate
Fabaceae	*Lathyrus roseus* Steven *	35.87 ± 1.97	29.60 ± 2.16	1.21	Prolate	Medium	Tricolporate	Reticulate–perforate
Fabaceae	*Melilotus officinalis* (L.) Desr.	25.06 ± 1.20	19.20 ± 0.94	1.31	Prolate	Small	Tricolporate	Reticulate–perforate
Fabaceae	*Onobrychis altissima* Grossh.	34.22 ± 2.79	20.75 ± 2.83	1.65	Prolate	Medium	Tricolpate	Suprareticulate
Fabaceae	*Securigera orientalis* (Mill.) Lassen *	20.48 ± 1.44	17.99 ± 1.57	1.14	Prolate	Small	Tricolporate	Rugulate–perforate
Fabaceae	*Securigera varia* (L.) Lassen	25.20 ±2.19	19.83 ± 2.36	1.27	Prolate	Small	Tricolporate	Rugulate–perforate
Fabaceae	*Trifolium ambiguum* M.Bieb. *	24.97 ± 1.11	19.60 ± 1.28	1.27	Prolate	Small	Tricolporate	Reticulate–perforate
Fabaceae	*Trifolium canescens* Willd. *	42.69 ± 4.66	35.83 ± 4.48	1.19	Prolate	Medium	Tricolporate	Reticulate–perforate
Fabaceae	*Trifolium pratense* L.	36.42 ± 3.52	32.41 ± 3.29	1.12	Prolate	Medium	Tricolporate	Reticulate–perforate
Fabaceae	*Trifolium spadiceum* L. *	21.74 ± 1.87	20.89 ± 1.68	1.04	Spheroidal	Small	Tricolporate	Psilate–perforate
Fabaceae	*Vicia balansae* Boiss. *	34.85 ± 1.86	23.90 ± 1.72	1.46	Prolate	Medium	Tricolporate	Verrucate–perforate
Fabaceae	*Vicia cracca* subsp*. cracca* L.	35.47 ± 2.40	23.50 ± 2.25	1.51	Prolate	Medium	Tricolporate	Verrucate–perforate
Gentianaceae	*Swertia iberica* Fisch. ex C.A.Mey. *	27.60 ± 1.36	26.24 ± 1.32	1.05	Spheroidal	Medium	Tricolporate	Striato–microreticulate
Geraniaceae	*Geranium ibericum* subsp*. jubatum* (Hand.–Mazz.) P.H.Davis * †	92.88 ± 4.18	84.79 ± 3.67	1.1	Prolate	Large	Tricolporate	Reticulate–clavate
Geraniaceae	*Geranium ponticum* (P.H.Davis & J.Roberts) Aedo * †	65.59 ± 7.19	60.99 ± 6.29	1.08	Prolate	Large	Tricolporate	Reticulate–clavate
Geraniaceae	*Geranium pyrenaicum* Burm.f.	56.72 ± 2.66	52.55 ± 2.19	1.08	Prolate	Large	Tricolporate	Reticulate–clavate
Lamiaceae	*Ajuga orientalis* L.	23.47 ± 2.32	19.04 ± 1.30	1.23	Prolate	Small	Tricolpate	Granulate
Lamiaceae	*Mentha longifolia* (L.) L.	23.68 ± 2.22	21.25 ± 1.87	1.11	Prolate	Small	Hexacolpate	Microreticulate
Lamiaceae	*Salvia verticillata* L.	21.26 ± 2.93	19.88 ± 2.71	1.07	Prolate	Small	Hexacolpate	Microreticulate–reticulate–bireticulate
Lamiaceae	*Stachys macrantha* (K.Koch) Stearn *	30.82 ± 3.32	27.17 ± 4.01	1.13	Prolate	Medium	Tricolpate	Reticulate–perforate
Lamiaceae	*Teucrium chamaedrys* L.	23.81 ± 2.14	23.81 ± 2.26	1	Spheroidal	Small	Tricolpate	Verrucate–perforate
Lamiaceae	*Thymus praecox* Opiz	25.37 ± 2.01	27.41 ± 2.86	0.93	Oblate	Medium	Hexacolpate	Bireticulate
Onagraceae	*Epilobium angustifolium* L.	48.65 ± 4.74	64.16 ± 4.03	0.76	Oblate	Large	Triporate	Microgemmate–perforate
Papaveraceae	*Papaver lateritium* K.Koch *†	22.46 ± 1.93	17.82 ± 3.00	1.26	Prolate	Small	Tricolpate	Microechinate–perforate
Plantaginaceae	*Digitalis ferruginea* L. *	20.14 ± 1.28	18.26 ± 1.40	1.1	Prolate	Small	Tricolporate	Microreticulate
Plantaginaceae	*Plantago lanceolata* L.	22.96 ± 1.03	22.38 ± 1.06	1.03	Spheroidal	Small	Pantoporate	Verrucate–microechinate
Polygonaceae	*Polygonum alpinum* All.	30.17 ± 1.77	26.90 ± 1.98	1.12	Prolate	Medium	Tricolpate	Microechinate–perforate
Polygonaceae	*Polygonum bistorta* subsp*. carneum* (K.Koch) Coode & Cullen	43.16 ± 4.37	32.70 ± 3.23	1.32	Prolate	Medium	Tricolporate	Microechinate–perforate
Polygonaceae	*Rumex alpinus* L.	15.06 ± 1.35	13.96 ± 1.69	1.08	Prolate	Small	Tricolporate	Microechinate–perforate
Ranumculaceae	*Anemone narcissiflora* L.	25.91 ± 1.83	21.70 ± 2.10	1.19	Prolate	Medium	Tricolpate	Microechinate–perforate
Ranumculaceae	*Caltha palustris* L.	27.34 ± 2.06	25.71 ± 2.84	1.06	Spheroidal	Medium	Tricolpate	Microechinate–perforate
Ranumculaceae	*Trollius ranunculinus* (Sm.) Stearn	20.98 ± 2.96	20.77 ± 3.79	1.01	Spheroidal	Small	Tricolpate	Striate–perforate
Rosaceae	*Geum aleppicum* Jacq. *	25.63 ± 3.21	22.50 ± 2.98	1.14	Prolate	Medium	Tricolporate	Striate–perforate
Rosaceae	*Potentilla crantzii* (Crantz) Fritsch *	20.60 ± 1.45	19.62 ± 2.34	1.05	Spheroidal	Small	Tricolporate	Striate–perforate
Rosaceae	*Prunus divaricata* Ledeb. *	30.69 ± 2.03	27.30 ± 2.97	1.12	Prolate	Medium	Tricolporate	Striate–perforate
Rosaceae	*Rosa canina* L.	24.68 ± 2.35	18.60 ± 2.08	1.33	Prolate	Small	Tricolporate	Striate–perforate
Rosaceae	*Rubus idaeus* L.	21.03 ± 1.46	18.10 ± 1.69	1.16	Prolate	Small	Tricolporate	Striate–perforate
Rubiaceae	*Galium verum* L.	18.81 ± 1.99	17.10 ± 2.36	1.1	Prolate	Small	Stephanocolpate	Microechinate–perforate

**Table 3:** Summary of the GLMM analysis of the selected melliferous plant species.

Index	Coef (*β*)	SE	z	*p*-Value	95% CI Lower	95% CI Upper	Var (Species)	R^2^ Marginal	R^2^ Conditional
Intercept	17.07	1.17	14.65	<0.001	14.78	19.35	65.61	0.4641	0.9621
Equatorial Diameter	0.50	0.02	25.08	<0.001	0.46	0.53	65.61	0.4641	0.9621

## Data Availability

The original contributions presented in the study are included in the article, further inquiries can be directed to the corresponding author.
